# Large-Scale Conformational Changes of FhaC Provide Insights Into the Two-Partner Secretion Mechanism

**DOI:** 10.3389/fmolb.2022.950871

**Published:** 2022-07-22

**Authors:** Giuseppe Sicoli, Albert Konijnenberg, Jérémy Guérin, Steve Hessmann, Elise Del Nero, Oscar Hernandez-Alba, Sophie Lecher, Guillaume Rouaut, Linn Müggenburg, Hervé Vezin, Sarah Cianférani, Frank Sobott, Robert Schneider, Françoise Jacob-Dubuisson

**Affiliations:** ^1^ Laboratoire Avancé de Spectroscopie pour les Interactions, la Réactivité et l’Environnement (LASIRE), UMR CNRS 8516, Université de Lille, Lille, France; ^2^ BAMS Research Group, University of Antwerp, Antwerp, Belgium; ^3^ CNRS, INSERM, Institut Pasteur de Lille, Université de Lille, U1019-UMR9017-CIIL-Center for Infection and Immunity of Lille, Lille, France; ^4^ Laboratoire de Spectrométrie de Masse BioOrganique, Université de Strasbourg, CNRS, Strasbourg, France; ^5^ Infrastructure Nationale de Protéomique ProFI – FR 2048, Strasbourg, France; ^6^ CNRS EMR9002 Integrative Structural Biology, Lille, France; ^7^ INSERM, CHU Lille, U1167 - RID-AGE - Risk Factors and Molecular Determinants of Aging-Related Diseases, Institut Pasteur de Lille, Université de Lille, Lille, France; ^8^ Astbury Centre for Structural Molecular Biology and the School of Molecular and Cellular Biology, University of Leeds, Leeds, United Kingdom

**Keywords:** outer membrane protein, protein dynamics, Omp85 superfamily, NMR, EPR, mass spectrometry, Gram-negative bacteria, two-partner secretion system

## Abstract

The Two-Partner secretion pathway mediates protein transport across the outer membrane of Gram-negative bacteria. TpsB transporters belong to the Omp85 superfamily, whose members catalyze protein insertion into, or translocation across membranes without external energy sources. They are composed of a transmembrane β barrel preceded by two periplasmic POTRA domains that bind the incoming protein substrate. Here we used an integrative approach combining *in vivo* assays, mass spectrometry, nuclear magnetic resonance and electron paramagnetic resonance techniques suitable to detect minor states in heterogeneous populations, to explore transient conformers of the TpsB transporter FhaC. This revealed substantial, spontaneous conformational changes on a slow time scale, with parts of the POTRA2 domain approaching the lipid bilayer and the protein’s surface loops. Specifically, our data indicate that an amphipathic POTRA2 β hairpin can insert into the β barrel. We propose that these motions enlarge the channel and initiate substrate secretion. Our data propose a solution to the conundrum how TpsB transporters mediate protein secretion without the need for cofactors, by utilizing intrinsic protein dynamics.

## Introduction

The Two-Partner Secretion (TPS) pathway is dedicated to the export of large proteins notably serving as virulence factors (Guérin et al., 2017). The TpsB transporters are transmembrane β-barrel proteins that secrete their substrates, collectively called TpsA proteins, across the outer membrane of various Gram-negative bacteria. They belong to the ubiquitous Omp85 superfamily whose members mediate protein insertion into, or translocation across membranes of bacteria and eukaryotic organelles, and which includes the essential bacterial BamA insertases (Knowles et al., 2009; Heinz and Lithgow, 2014; Noinaj et al., 2017). The FhaB/FhaC pair of *Bordetella pertussis* is a model TPS system, in which the FhaC transporter is necessary and sufficient to mediate the translocation of the adhesin FhaB across the outer membrane (Fan et al., 2012).

Omp85 proteins are composed of N-terminal POTRA (polypeptide transport associated) domains - two in the case of TpsB transporters - followed by a 16-stranded transmembrane β barrel, which for FhaC is the FhaB translocation pore (Baud et al., 2014). The POTRA domains mediate protein-protein interactions in the periplasm, and notably recognition of client proteins (Delattre et al., 2011). Another hallmark feature of the Omp85 superfamily is the extracellular loop L6 that folds back inside the barrel and harbors a conserved motif at its tip forming a salt bridge interaction with a specific motif of the inner β-barrel wall (Gu et al., 2016; Maier et al., 2015; Noinaj et al., 2013).

**FIGURE 1:**
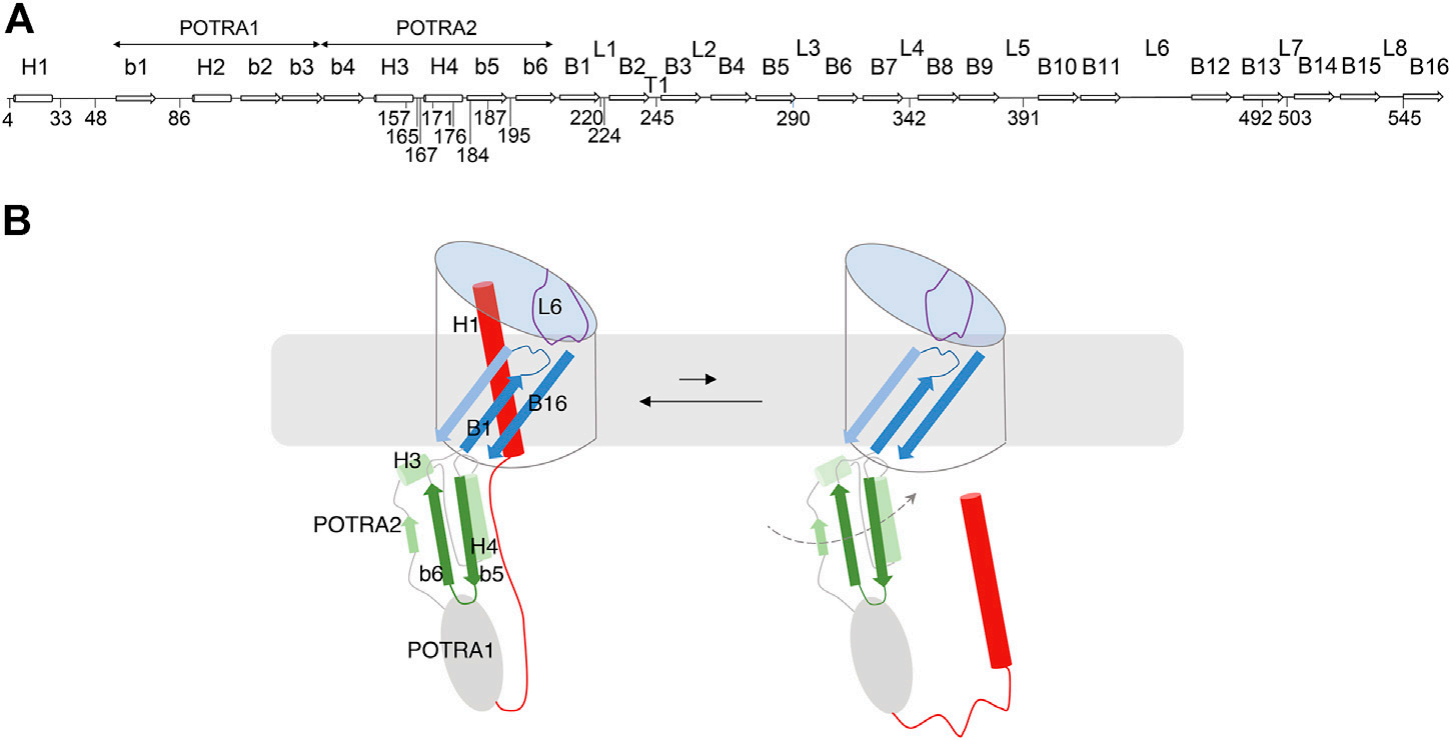
FhaC: Secondary structure and intrinsic dynamics. **(A)** Linear representation of the secondary structure elements of FhaC, with residues used in this work. L1 to L8 represent the extracellular loops, b1 to b6 the β strands of the POTRA domains, H1 to H4 the α helices, and B1 to B16 the β-barrel strands. T1 is the first periplasmic turn. **(B)** Schematic representation of known FhaC motions linked to its activity. Specific structural elements are colored as follows: H1 and the linker in red, the POTRA2 domain in shades of green, L6 in purple, and β barrel strands B1, B2 and B16 in shades of blue. In its resting conformation (left), FhaC is closed, with H1 crossing the β barrel. The linker is positioned along the H4 helix of the POTRA2 domain (Maier et al., 2015), thus obstructing a major substrate binding site (Delattre et al., 2011). L6 is folded inside the barrel with a conserved interaction between its tip and the barrel wall. This conformation is in equilibrium with an open form (right), in which H1 has vacated the pore and the substrate binding site is available (Guérin et al., 2014). We have obtained evidence that L6 also moves away from its resting position in the β barrel and that the POTRA2 domain undergoes so far undefined motions (represented as a dashed line) (Guérin et al., 2015).

A specific feature of TpsB transporters is an N-terminal α helix called H1 that plugs the β barrel (Clantin et al., 2007; Guérin et al., 2014; Maier et al., 2015; Guérin et al., 2020) (Figure 1A,B). An extended linker follows H1 and joins it to the POTRA1 domain in the periplasm. Recently, the X-ray structures of the TpsB transporters CdiB^Ab^ and CdiB^Ec^ have shown very similar folds to that of FhaC, albeit with slightly different positions of H1 in the barrel (Guérin et al., 2020). Both H1 and L6 stabilize the barrel in a closed conformation that most likely corresponds to the resting state of the transporter (Maier et al., 2015; Guérin et al., 2020). The β barrel, the L6 loop, and the two POTRA domains are essential for transport activity (Clantin et al., 2007).

Omp85 proteins likely function in the absence of ATP or an electrochemical gradient. They appear to be very dynamic and to undergo conformational cycling (Renault et al., 2011; Iadanza et al., 2016; Doerner and Sousa, 2017; Warner et al., 2017; Guérin et al., 2020). Lateral opening of the barrel between the first and last anti-parallel β strands is a common mechanistic feature of Omp85 insertases (Noinaj et al., 2014; Estrada Mallarino et al., 2015; Iadanza et al., 2016; Höhr et al., 2018; Doyle and Bernstein, 2019; Diederichs et al., 2020; Tomasek et al., 2020). A partial lateral opening of the barrel seam was also shown to be important for the function of a TpsB transporter (Guérin et al., 2020).

The mechanism of two-partner secretion remains poorly understood, but it is known to involve substantial conformational changes of the transporter including exit of H1 from the β barrel and motions of the L6 loop (Guérin et al., 2014; Guérin et al., 2015; Guérin et al., 2020) (Figure 1B). The motion of H1 toward the periplasm is facilitated by conformational changes of flexible regions of the barrel, in particular the first β-barrel strand B1 and the extracellular loops L1, L2 and L6 (Guérin et al., 2020). Binding of the conserved N-terminal ‘TPS’ domain of the substrate protein to the POTRA domains of its transporter also appears to enhance conformational changes (Guérin et al., 2015; Guérin et al., 2020). How the substrate enters the pore and is progressively hoisted towards the cell surface without backsliding to the periplasm remains unknown, but we hypothesize a mechanism implying yet uncharacterized transient conformations of TpsB transporters. In this work we have explored such FhaC conformers using biophysical techniques suitable to detect minor states in heterogeneous populations. Our data revealed spontaneous changes of the conformation of the POTRA2 domain and of its position relative to the barrel, on a slow time scale.

## Materials and Methods

### Strains and Plasmids

*Escherichia coli* JCB570 (parent) or JCB571 (*dsbA*::kan) (Bardwell et al., 1991) were used for low level expression of FhaC and *E. coli* BL21 (DE3*-omp5*) (*ΔlamB ompR*) (Prilipov et al., 1998) for overexpression. Point mutations in *fhaC* were generated using the QuikChange II XL Kit (Agilent, Les Ulis, France) on pFc3 (Guédin et al., 2000). Overexpression of FhaC for purification was performed from pET22 or pET24 plasmids (Clantin et al., 2007). ptacFha44-His codes for the first 80 kDa of the FhaC substrate FhaB, called Fha44, followed by a 6-His tag. It was constructed by adding a 1.2-kb Sal-BamHI fragment of the *fhaB* gene into the same sites of ptacNM2lk-His (Guérin et al., 2015). pFJD63 codes for FhaC under the control of the P_BAD_ promoter (Guédin et al., 1998). Its derivatives were constructed by ligating the XhoI-HindIII and XhoI-XbaI fragments of pFJD63 with the XbaI-HindIII *fhaC* fragments carrying the relevant mutations from the pFc3 derivatives. pMSP1D1 and pMSP1E3D1 were obtained from Addgene (Watertown, MA, USA). pSphB1αβ is a derivative of pT7SB1αβ-S (Dé et al., 2008) with a 6-His tag. To construct pT7bfrG-H, the sequence corresponding to the mature protein was PCR amplified and inserted in pFJD138 (Méli et al., 2006) after the signal-peptide and 6-His tag sequences.

### *In vivo* Assays

To monitor disulfide (S-S) bond formation *in vivo*, the pFc3 variants were introduced in *E. coli* JCB571. The recombinant bacteria were grown at 37°C in minimum M9 medium containing 0.1% casaminoacids under agitation. The cells were collected by centrifugation when the optical densities at 600 nm (OD_600_) of the cultures reached 0.8. The cell pellets were resuspended in 50 mM sodium phosphate (pH 6.8) containing 10 mM N-ethylmaleimide and lysed using a Hybaid ribolyzer apparatus (50 s at speed 6). The membranes were collected by ultracentrifugation of the clarified lysates at 90,000 g for 1 h. The pellets were resuspended in loading buffer without reducing agent and separated into two aliquots, with dithioerythritol (DTE) added at 25 mM to one of them before heating at 70 °C for 10 min. FhaC was detected using anti-FhaC antibodies (Delattre et al., 2011) with alkaline phosphatase development for 15 min.

For the secretion assays, overnight cultures of *E. coli* JCB570 or JCB571 harboring a pFJD63 derivative and ptacFha44-His were diluted to OD_600_ of 0.3 in LB and grown under agitation with 0.1% arabinose for 20 min to produce FhaC. The bacteria were collected by centrifugation, resuspended in prewarmed LB without arabinose and grown to OD_600_ of 0.8 before adding IPTG at 1 mM to induce the expression of Fha44. Culture aliquots were collected 5 and 20 min thereafter and placed on ice. After centrifugation to pellet the bacteria, Fha44 was affinity-purified from the supernatants with Ni-NTA beads (Qiagen, Courtaboeuf, France). The membrane extracts were prepared and FhaC was detected as above. Fha44 was detected by immunoblotting using anti-6His antibodies, the ECL kit of Amersham (Merck, St Quentin-Fallavier, France) and the Amersham Imager 600 (GE) with 1 s exposure. The amounts of Fha44 in supernatants were quantified with ImageJ.

### Protein Purification and Spin Labeling

The production and purification of the FhaC derivatives and of Fha30^N66I^ were performed as described (Guérin et al., 2014). Expression for nuclear magnetic resonance (NMR) experiments was performed in M9 minimal medium in D_2_O, 2.5 g/L ^2^H-glucose (Sigma, St Quentin-Fallaviers, France), 1 g/L^15^N-NH4Cl, 1 g/L^15^N,^2^H-isogro (Sigma) and ^13^C-α-ketobutyric acid (Sigma) to achieve u-(^2^H,^15^N), Ile-δ_1_(^13^CH_3_) isotope labeling (Ruschak and Kay, 2010). For spin labeling, 3 mM tris(2-carboxyethyl)phosphine (TCEP, Sigma) was added to the detergent extract before ion exchange chromatography. The FhaC-containing fractions were mixed with a 10-fold molar excess (1-oxyl-2,2,5,5-tetramethyl-Δ3-pyrroline-3-methyl) methanethiosulfonate (MTSL) or its diamagnetic analogue (1-Acetoxy-2,2,5,5-tetramethyl-δ-3-pyrroline-3-methyl) methanethiosulfonate (Toronto Research Chemicals, North York, ON, Canada) at 15 °C with gentle agitation for 16 h. Excess MTSL was removed by chromatography. SphB1-αβ and BfrG were produced from *E. coli* BL21 (DE3*-omp5*) and purified from β-octylglucoside (bOG) extracts using Ni^2+^ affinity chromatography. For BfrG 300 mM NaCl was added to improve solubility.

### Preparation of Liposomes and Nanodiscs and Protein Reconstitution

Small unilamellar vesicles (SUVs) of *E. coli* polar lipids were prepared as described (Guérin et al., 2014). Where indicated, SUVs were also prepared using mixtures of dimyristoyl phosphatidyl choline (DMPC) and dimyristoyl phosphatidyl glycerol (DMPG) (Avanti, Interchim, Montluçon, France). The SUVs were mixed with FhaC variants at lipid:protein molar ratios of approx. 2500:1 for electron paramagnetic resonance (EPR) and 200:1 for NMR experiments, respectively, at room temperature, with gentle agitation for 1 hour. The proteoliposomes were formed by removal of detergent with the progressive addition of Biobeads SM2 (Bio-Rad), and the proteoliposomes were collected by ultracentrifugation. All steps were performed under argon. Final buffer concentrations after mixing FhaC and liposomes were about 12.5 mM each of Tris-HCl and NaP_i_, 150 mM NaCl, pH 6.7.

Nanodiscs were prepared with the MSP1D1 and MSP1E3D1 scaffold proteins (Ritchie et al., 2009) produced in *E. coli* BL21 (DE3), with an induction of 3 h at 28 °C. For NMR experiments, scaffold proteins were expressed in M9 minimal medium in D_2_O using ^2^H-glucose as carbon source to suppress their signals in the (^1^H,^13^C)-based NMR spectra. The bacteria were broken using a French press in 50 mM Tris-HCl (pH 8), 300 mM NaCl (TN buffer), 1% Triton X100 (TNX buffer), and the clarified lysates were subjected to Ni^2+^ affinity chromatography. After successive washes in TNX, TN buffer with 50 mM cholate, 20 and 50 mM imidazole, the proteins were eluted in TN buffer with 400 mM imidazole, concentrated by ultrafiltration and dialyzed against 20 mM Tris-HCl (pH 8), 200 mM NaCl and 0.1 mM EDTA. DMPC and DMPG at a 2:1 ratio were solubilized in chloroform, lyophilized overnight and resuspended to 25 mM in 20 mM Tris-HCl (pH 7.5), 100 mM NaCl, 0.5 mM EDTA, 50 mM cholate. For NMR experiments, deuterated (d_54_-) DMPC and DMPG (Cortecnet, Voisins-le-Bretonneux, France) were used. FhaC, the scaffold protein and the lipids were mixed at a ratio of 1:3:180, cholate was added to 15 mM, and incubation was performed for 1 h at room temperature. Biobeads were added progressively, and the incubation was continued at 4 °C overnight. The nanodiscs were collected by ultracentrifugation and concentrated by ultrafiltration. For NMR experiments, the buffer was exchanged to 100 mM NaP_i_ in D_2_O pH* 7.2 using a 2-ml ZebaSpin column (7 kDa MWCO).

### NMR Experiments

For solid-state NMR experiments on FhaC variants reconstituted into liposomes, the proteoliposomes collected by ultracentrifugation were transferred to 1.3 mm magic-angle-spinning (MAS) solid-state NMR rotors (Bruker Biospin, Ettlingen, Germany) using an ultracentrifugation device (Bertini et al., 2012) (Giotto Biotech, Sesto Fiorentino, Italy) in a Beckman ultracentrifuge (SW 32 Ti rotor, 77,000 x g, 12°C, 30–60 min). NMR experiments were performed on spectrometers operating at 800 and 950 MHz ^1^H Larmor frequency (18.8 and 22.3 T magnetic field) (Bruker Biospin) at a MAS frequency of 50 kHz. Sample temperature was kept at about 17°C as judged by the chemical shift of the bulk water resonance. Spectra were indirectly referenced to 2,2-dimethyl-2-silapentane-5-sulfonate (DSS) via the lipid methylene proton resonance, which appears at 1.225 ppm under our experimental conditions. Typical pulse lengths for ^1^H and ^13^C 90° pulses were 2.1 and 3.8 µs, respectively. For cross-polarization (CP), radiofrequency (RF) field strengths were 21 and 30 kHz for ^1^H and ^13^C, respectively, with a 50-to-100% ramp on ^1^H and a duration of 1.5 ms. ^1^H-detected 2D ^13^C-^1^H dipolar hCH correlation spectra (Barbet-Massin et al., 2014) were typically recorded with 1600 data points and a spectral width of 40 ppm in the direct ^1^H dimension and 100 to 140 data points and a spectral width of 13 ppm in the indirect ^13^C dimension. For water suppression, the MISSISSIPPI scheme (Zhou and Rienstra, 2008) was used (15 kHz ^1^H RF). For the 2D hChH correlation spectrum, a ^1^H–^1^H mixing time of 6.4 ms using radio frequency driven recoupling (RFDR) (Bennett et al., 1992) was applied between back-CP and acquisition (^1^H RF 120 kHz). ^13^C R_1ρ_ spectra (Lewandowski et al., 2011; Ma et al., 2014) were recorded with ^13^C spinlock field strengths from 1.2 to 10 kHz (applied on-resonance with isoleucine δ_1_^13^C methyls) and 5 spinlock durations from 2.5 to 80 ms, with a ^1^H 180° pulse in the middle of the spinlock to suppress chemical shift anisotropy/dipolar coupling cross-correlated relaxation (Kurauskas et al., 2016). For solid-state paramagnetic relaxation enhancement (PRE) NMR experiments, dipolar 2D hCH correlation spectra were recorded on FhaC single Cys mutants Cys^187^ and Cys^220^ bearing a paramagnetic MTSL tag (FhaC^187R1^, FhaC^220R1^), reconstituted into *E. coli* polar lipid liposomes. These spectra were referenced to spectra recorded on FhaC^187R1^ after reduction of MTSL by a 3.5-fold molar excess of ascorbic acid (FhaC^187R1red^) and FhaC^C220^ carrying a diamagnetic MTSL analogue, respectively (Battiste and Wagner, 2000; Nadaud et al., 2007).

Solution-state NMR experiments on FhaC in nanodiscs were conducted on a 900 MHz spectrometer (Bruker Biospin) at 32°C sample temperature. Standard ^13^C-^1^H heteronuclear multiple-quantum coherence (HMQC) or SOFAST-HMQC (Schanda and Brutscher, 2005) experiments were recorded with 2048 and 150 data points and spectral widths of 14 and 7.4 ppm in direct ^1^H and indirect ^13^C dimensions, respectively. For PRE experiments, SOFAST-HMQC spectra were recorded on a FhaC^195R1^ sample before and after reduction of the paramagnetic MTSL tag with a 10-fold molar excess of ascorbic acid (Battiste and Wagner, 2000).

NMR spectra were processed with TopSpin 4.0.3 (Bruker Biospin) or NMRPipe (Delaglio et al., 1995) and analyzed with Sparky (Lee et al., 2015) or CcpNMR (Vranken et al., 2005).

For assignment of isoleucine (Ile) δ_1_ methyl signals, Ile-to-Val mutations were used in all cases except for Ile^114^ in domain POTRA1, which was identified by attenuation of its signal in the spectrum of paramagnetic FhaC^195R1^ (average distance of Ile^114^ Cδ_1_ to the paramagnetic center modeled on residue 195: 16.6 Å), and Ile^420^, which was assigned by exclusion.

To generate relaxation dispersion curves, effective transverse relaxation rates *R*_2,eff_ were extracted from experimental *R*_1ρ_ values using separately recorded *R*_1_ experiments (Palmer and Massi, 2006). *R*_2,eff_ error estimates were obtained by error propagation from estimates of the errors in spectral intensities based on repeat experiments. F tests were used to assess whether a model assuming exchange (Davis et al., 1994) fitted relaxation dispersion curves significantly better than a model assuming no exchange (i.e., constant *R*_2,eff_).

For PRE NMR experiments, ratios of peak intensities in spectra of para- and diamagnetic species (FhaC^220R1^/FhaC^220R1dia^ and FhaC^187R1^/FhaC^187R1red^ for solid-state experiments, FhaC^195R1^/FhaC^195R1red^ in case of the solution-state experiments, respectively) were calculated. These para-versus diamagnetic signal intensity ratios do not normalize to one in our case. In the solid-state experiments, this can be explained by different amounts of protein integrated into liposomes during reconstitution (FhaC^220R1^ vs FhaC^220R1dia^), as well as different amounts of sample transferred to the NMR rotor (FhaC^220R1^ vs FhaC^220R1dia^ as well as FhaC^187R1^ before and after reduction). Both in solid and solution state, spectroscopic factors also play a role (incomplete longitudinal relaxation and thus lower signal-to-noise in the spectra of diamagnetic samples due to the use of short inter-scan delays (Iwahara et al., 2007)). For the solid-state experiments, we have thus opted to normalize PRE ratios to the maximum ratio observed in each experiment, which was always observed in one of the residues furthest from the paramagnetic center according to the FhaC crystal structure (Ile^136^ in FhaC^220R1^, Ile^441^ in FhaC^187R1^). This is equivalent to normalizing signals within each spectrum to a reference signal whose intensity is unaffected by PRE effects. We then only analyzed relative signal attenuation levels, instead of attempting to extract quantitative distance measures. Error bars of PRE intensity ratios were calculated based on the root-mean-standard deviation of the spectral noise using standard error propagation. To test for significant differences between these intensity ratios, *p* values for comparisons were calculated from *z* scores, assuming errors of intensity ratios to be normally distributed. To estimate expected distances between FhaC Ile residues and the MTSL label, and consequently relative PRE attenuation levels, ensembles of 200 MTSL conformations compatible with labeling on FhaC residues 187, 195, and 220 were calculated using the mtsslSuite web server or PyMOL plugin (Hagelueken et al., 2012; Hagelueken et al., 2015) (http://www.mtsslsuite.isb.ukbonn.de/) and the FhaC crystal structure (PDB 4QKY) with the corresponding residue changed to Cys in PyMOL (The PyMOL Molecular Graphics System, Schrödinger, LLC; https://pymol.org/). Distances from the average position of the paramagnetic center as given by mtsslSuite to Ile Cδ1 nuclei were calculated using PyMOL.

### EPR Experiments

Continuous-wave (CW) EPR experiments were performed as described (Guérin et al., 2014). Pulsed-electron double-resonance (PELDOR) spectroscopy experiments were performed at Q-band frequency (∼34 GHz) using a Bruker EleXsys E580 spectrometer equipped with an overcoupled Bruker EN 5107D2 resonator. Pulses were generated with a Bruker SpinJet AWG and amplified with a 50 W TWT amplifier. The experiments were performed at 50 and 30 K using a variable-temperature cryogen-free system (Oxford, Oxford, UK). The deadtime-free, four-pulse PELDOR sequence [(π/2)probe ― τ1 ― (π)probe ― τ1 + *t* ― (π)pump ― τ2 –t ― (π)probe ― τ2 ― (echo)] was employed with a 200-ns τ1 delay and τ2 delays ranging from 3,200 ns to 7,000 ns depending on the sample (Pannier et al., 2000). Probe pulses were 10 ns (π/2) and 20 ns (π) Gaussian-shaped pulses at a frequency corresponding to the maximum of the resonator response function and a magnetic field value corresponding to the high-field shoulder of the echo-detected field-swept spectrum. The pump pulse was implemented as a 24-ns pulse centered at a frequency 55 MHz higher than the probe frequency and corresponding to the maximum of the nitroxide field-swept spectrum. Raw time-domain PELDOR traces were background-corrected using the DeerAnalysis 2019 package (Jeschke et al., 2006), and the resulting signals were power-scaled in MATLAB to suppress sum and difference peaks arising from multispin effects. Distance distributions were then calculated from the scaled and background-corrected PELDOR traces by Tikhonov regularization. For FhaC^33R1+503R1^, FhaC^187R1+503R1^ and FhaC^195R1+503R1^, distance distributions were predicted using a pre-computed rotamer library of the MTSL spin probe attached to specific residues on the PDB structure (Jeschke, 2021).

### Native Mass Spectrometry (MS) and Ion Mobility

Purified FhaC was buffer exchanged into 100 mM ammonium acetate buffer, pH 6.8, supplemented with 50 mM bOG using a P6 desalting column (Biorad, Marnes-la-Coquette, France). Samples were directly infused with nano-electrospray ionization with in-house-prepared gold-coated borosilicate glass capillaries with a spray voltage of +1.4 kV. Spectra were recorded on a quadrupole TOF instrument (Synapt G2 HDMS with 32K quadrupole, Waters) optimized for transmission of native, high-m/z protein assemblies. Critical voltages and pressures throughout the instrument were 50, 10, 150 and 15 V for the sampling cone, extraction cone, trap and transfer collision cell, respectively, with pressures of 9 mbar, 1.47 × 10^–2^ mbar and 1.21 × 10^–2^ mbar for the source, trap and transfer regions unless indicated otherwise. Collision-induced unfolding (CIU) ion mobility experiments were performed with 50 V sampling cone; 50–200 V trap collision energy; 42 V trap DC bias; and 15 V transfer collision energy. Pressures throughout the instrument were 9 and 1.46 × 10^–2^ mbar for the source and trap/transfer collision cells. All spectra were processed with Masslynx v4.1 (Waters). Collision cross section (CCS) calibration was performed using GDH, ADH, ConA and PK as proteins standard as described (Allen et al., 2016). It should be noted that due to the generally lower charge states observed for membrane proteins, and the increased collision energies required (compared to soluble proteins) for gentle release of proteins from detergent micelles, the CCS values reported here are less accurate and intended for qualitative comparison rather than quantitative matching to theoretical models.

### Peptide Binding Assays

Synthesized peptides were dissolved in DMSO to a final concentration of 100 mM and added to the protein sample at final concentrations of 10 μΜ FhaC and 100 μM peptide. To correct for non-specific and detergent-specific binding, SphB1-αβ was run at identical concentrations and conditions. For both proteins the fraction of peptide-bound protein was calculated based on peak intensities, after which the binding to the decoy protein was subtracted to correct for non-specific binding.

## Results

### *In vivo* Evidence for Alternative Conformations of the POTRA2 Domain

We previously obtained hints that motions of the H1 helix and the L6 loop affect the conformation of the POTRA2 domain (Guérin et al., 2015; Guérin et al., 2020). To obtain insight into its putative alternative conformers, we simultaneously replaced two residues distant in the ‘resting conformation’ of FhaC (i.e., its X-ray structure) with Cys residues in order to detect spontaneous S-S bond formation. Of note, FhaC is naturally devoid of Cys residues. Our rationale was that conformational changes that bring the two Cys residues close to each other should promote intramolecular S-S bond formation even if the corresponding conformations are short-lived, as the S-S bound species accumulate over time. S-S bond formation, which is catalyzed by the periplasmic disulfide oxidase DsbA in the course of biogenesis, generally affects SDS-PAGE migration in the absence of a reducing agent. These experiments were thus performed in a *dsbA* ^*-*^ background, such that S-S bonds could spontaneously form after FhaC biogenesis as a result of its conformational changes in the membrane.

**FIGURE 2:**
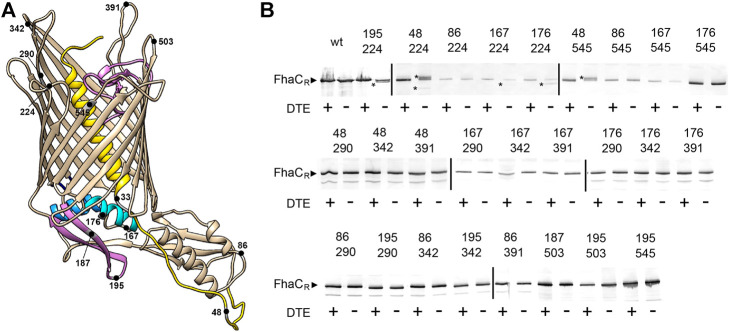
Detection of transient conformers of FhaC *in vivo*. **(A)** Structural model of FhaC (PDB 4QKY) and positions of the Cys substitutions. Helix H1 and loop L6 are drawn in yellow and purple, respectively; POTRA2 helix H4 and hairpin b5-b6 are in cyan and purple. **(B)** Immunoblot of membrane fractions of *E. coli JCB571* (*dsbA* KO strain) producing FhaC variants. The numbers indicate the positions of the two Cys residues. The reducing agent DTE was added to one half of each sample. FhaC_R_ represents the position of the reduced form. The asterisks point to the additional, intramolecular cross-linked forms that can migrate faster or more slowly than the reduced form, depending on the respective positions of the two Cys residues. At least three independent replicate experiments (cultures and blots) were performed for all variants. Note that the fuzzy band for FhaC(Cys167 + Cys342) in the +DTE lane, which was not reproducibly observed, does not correspond to a S-S-bonded species, since it was found only in the presence of the reducing agent.

We combined Cys residues at the extracellular surface at positions 224 in L1, 290 in L3, 342 in L4, 391 in L5, 503 in L7 and 545 in L8 with periplasmic Cys residues in the POTRA2 domain at positions 167, 176, 187, and 195, in the linker at position 48, and in the POTRA1 domain at position 86 (Figure 2A). None of the single Cys substitutions markedly affected the secretion activity of FhaC (Baud et al., 2014; Guérin et al., 2014; Guérin et al., 2015). Under non-reducing conditions, aberrant migration of FhaC in SDS-PAGE was identified for the combinations Cys^195^ + Cys^224^, Cys^176^ + Cys^224^, Cys^48^ + Cys^224^, Cys^48^ + Cys^545^ and weakly for Cys^167^ + Cys^224^, indicating S-S bond formation within specific pairs of engineered Cys residues (Figure 2B). For FhaC(Cys^48^ + Cys^224^), in the absence of reducing agent, we reproducibly obtained two species migrating above and below the reduced form, probably corresponding to different conformations of the denatured cross-linked protein. In contrast, no loop other than L1 or L8 was found to cross-link with these periplasmic regions, and none cross-linked with the Cys residue in the POTRA1 domain. Thus, *in vivo*, the last portion of the H1-POTRA1 linker can be found close to the extracellular loops L1 and L8 that immediately follow and precede the first and last β−barrel strands, B1 and B16, respectively, and the b5-b6 β hairpin and the H4 helix of the POTRA2 domain can be found close to the extracellular loop L1, indicating that these periplasmic elements approach the β−barrel seam in specific conformers.

### Evidence for dynamics and alternative conformations of FhaC in lipid bilayers by NMR spectroscopy

**FIGURE 3:**
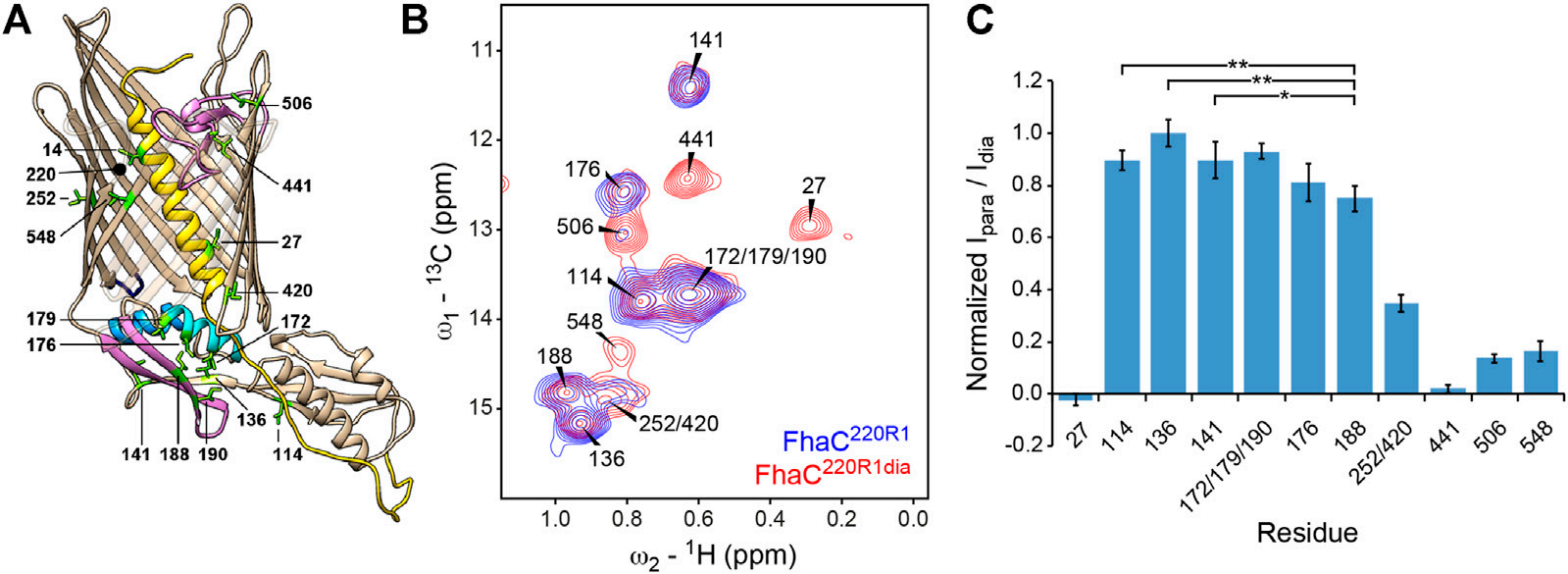
NMR analysis of Ile δ_1_-methyl labeled FhaC in lipid bilayers. **(A)** Structure of FhaC with Ile residues labeled and drawn as green sticks. Color code of structural elements is as in Figure 2. β-strands 12 to 15 are drawn transparently for visibility. The position of residue 220 used for PRE experiments is also indicated. **(B)** Superposition of the methyl regions of solid-state dipolar hCH ^13^C-^1^H correlation spectra of u-(^2^H, ^15^N), Ile-δ_1_(^13^CH_3_)-labeled FhaC samples in *E. coli* polar lipid liposomes, with a paramagnetic MTSL tag (FhaC^220R1^, blue) or with a diamagnetic MTSL analog (FhaC^220R1dia^, red) attached to the introduced Cys^220^ residue. Spectra were recorded at 800 MHz ^1^H Larmor frequency and 50 kHz MAS. **(C)** Ratios *I*_para_/*I*_dia_ of Ile-δ1 methyl peak intensities in the hCH correlation spectra of FhaC^220R1^ and FhaC^220R1dia^ shown in **(B)**, normalized to the maximum ratio observed in Ile^136^. Error bars are calculated based on spectral noise levels. * and ** indicate significant (*p* < 0.05 and *p* < 0.01, respectively) attenuation of the Ile^188^ signal relative to the signals of the reference residues Ile^114^, Ile^136^, and Ile^141^. See also Supplementary Figure S5.

To gain insight into the nature and the time scale of these conformational changes, we made use of NMR spectroscopy for its ability to characterize molecular structure and dynamics as well as minor conformational states of proteins in lipid bilayer environments (Mittermaier and Kay, 2009; Liang and Tamm, 2016). We recorded NMR spectra of FhaC in liposomes and lipid nanodiscs (Bayburt et al., 1998; Viegas et al., 2016), using solid- and solution-state NMR techniques, respectively. Both liposomes and nanodiscs provide a lipid bilayer environment for membrane proteins. However, while nanodiscs are disc-shaped lipid bilayer patches with diameters usually between 6 and 17 nm, held together by amphipathic apolipoproteins, liposomes are vesicles with diameters from tens of nanometers up to micrometers. Thus, proteins reconstituted into nanodiscs are amenable to study by solution NMR, while solid-state NMR methods are required for the study of proteins in liposomes. To render the 61-kDa FhaC protein more accessible to NMR spectroscopy, we resorted to perdeuteration and specific ^1^H, ^13^C-isotope labeling of isoleucine (Ile) δ_1_ methyl groups (Ruschak and Kay, 2010). Since the 15 Ile residues of FhaC are well distributed across all structural elements of the protein (Figure 3A), we expected this reduced labeling scheme to nevertheless be able to report on larger-scale structural transitions of FhaC.

Signals from all Ile residues could be identified and assigned by Ile-to-Val mutations or PRE experiments (Figure 3B; see Methods) (Amero et al., 2011; Venditti et al., 2011). The higher resolution of solution-state NMR spectra of FhaC in nanodiscs proved useful in the assignment (Supplementary Figure S1). Variable intensities of the Ile δ_1_ methyl signals in solid-state NMR experiments report on local dynamics in the protein in liposomes. While Ile^14^ in H1 was only visible in scalar coupling-based spectra, the signal of Ile^548^ in β-strand B16 at the barrel seam consistently exhibited low intensity in both scalar and dipolar coupling-based spectra (Figure 3B; Supplementary Figure S1). This indicates sub-µs time scale motion towards the N-terminus of the H1 helix and µs-to-ms time scale exchange dynamics at the barrel seam, respectively. The notion of dynamics in FhaC is also supported by the absence of through-space correlations for all but the shortest Ile-Ile distances expected from the crystal structure (Supplementary Figure S2). However, ^13^C rotating-frame (R_1ρ_) relaxation dispersion experiments probing exchange between states with different chemical shifts on the µs time scale (Lewandowski et al., 2011; Ma et al., 2014) yielded statistically flat dispersion profiles (Supplementary Figure S3), indicating that conformational changes of FhaC detectable by Ile δ_1_ methyl chemical shifts must occur on slower time scales.

To specifically probe for alternative FhaC conformations, we performed PRE NMR experiments in which a paramagnetic MTSL spin label is attached to an engineered Cys in the protein, and attenuation of NMR signals of nuclei within a radius of about 25–30 Å around the MTSL probe can be detected even if they only transiently approach the probe (Battiste and Wagner, 2000; Nadaud et al., 2007; Clore and Iwahara, 2009). To probe for conformers in which parts of the POTRA2 domain approach the extracellular surface, we chose residue 187 in the POTRA2 b5-b6 β hairpin and residue 220 at the beginning of the extracellular loop L1 for these experiments, yielding FhaC^187R1^ and FhaC^220R1^, where R1 represents the spin label. Intensities of Ile δ_1_ methyl signals in FhaC^187R1^ and FhaC^220R1^ measured by solid-state NMR in proteoliposomes were referenced to those measured in the same sample after reduction of the paramagnetic center with ascorbic acid (FhaC^187R1red^) or in a sample with a diamagnetic MTSL analog attached to the same residue (FhaC^220R1dia^), respectively (Figure 3B,C, Supplementary Table S4 and Supplementary Figure S5). While absolute quantification of distances from these data is difficult due to variations in the amounts of protein reconstituted into liposomes and transferred into solid-state NMR rotors, comparison of the para-versus diamagnetic signal intensity ratios *I*_para_/*I*_dia_ obtained for different FhaC residues allows to determine whether a signal is more attenuated than expected from the crystal structure, indicating a residue approaching the probe more closely (see Methods for details).

In FhaC^220R1dia^, in agreement with expectations, residues more than 35 Å away from the position of the paramagnetic MTSL tag modelled onto the FhaC crystal structure (Ile^114^, Ile^136^, and Ile^141^ in POTRA1 and POTRA2) exhibited the highest *I*_para_/*I*_dia_ ratios, while residues expected to be within 16–25 Å of the paramagnetic center (Ile^27^, Ile^441^, Ile^506^, Ile^548^) showed attenuation of their NMR signals (Figure 3B,C, Supplementary Table S4 and Supplementary Figure S5). The overlapped signal corresponding to residues Ile^252^ and Ile^420^, at expected distances to the paramagnetic center of 12 and 32 Å, respectively, exhibited intermediate attenuation. Interestingly, the signal of Ile^188^ in strand b5 of the POTRA2 domain was attenuated more than would be expected for a residue at 35 Å distance from the paramagnetic center. The difference in attenuation is significant with respect to the reference residues Ile^114^, Ile^136^, and Ile^141^ (*p* < 0.05, Figure 3C). Similarly, in FhaC^187R1^, the signal of Ile^506^ on the extracellular side of the barrel showed attenuation despite a distance of more than 50 Å to the MTSL tag modelled onto residue 187 of the FhaC crystal structure, while peaks due to residues Ile^172^, Ile^176^, and Ile^179^ in the POTRA2 helix H4 were still present in the spectrum despite being within 15 Å of the MTSL tag in the crystal structure (Supplementary Figure S5). These results support the notion that a region of the POTRA2 domain encompassing strand b5 can approach the extracellular loops of FhaC.

### Evidence for Motions of the POTRA2 Domain Towards the Extracellular Side by EPR Spectroscopy

To complement the NMR data, we resorted to EPR spectroscopy, another technique suitable to detect dynamics and minor conformational states of proteins in lipid bilayers (Liang and Tamm, 2016; Sahu and Lorigan, 2020). Distances from about 1.8 to 8 nm between paramagnetic spin labels attached to membrane proteins can be measured with PELDOR EPR experiments and can provide insight into non-homogeneous conformational ensembles (Jeschke, 2012).

**FIGURE 4:**
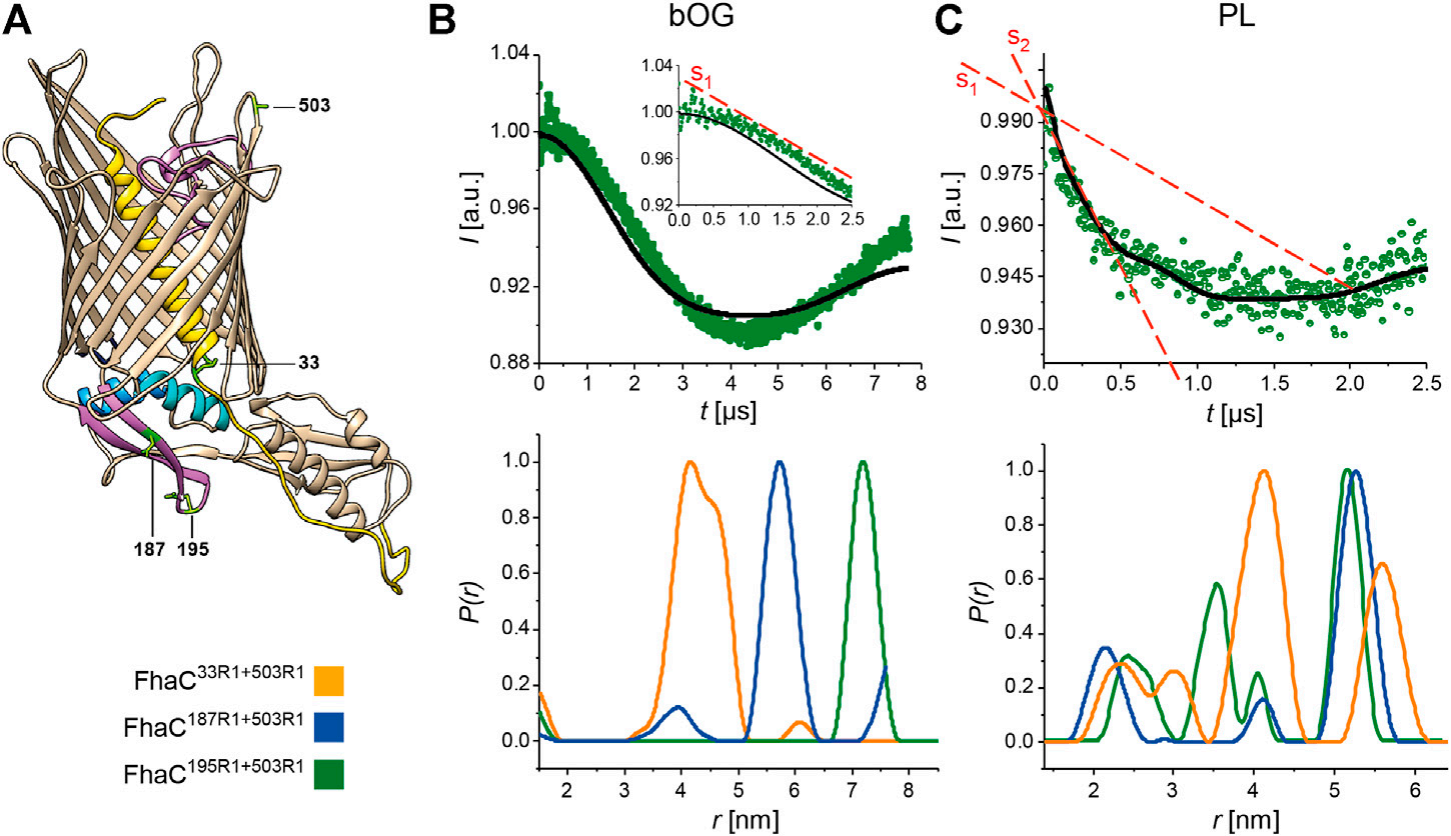
PELDOR analyses of FhaC. **(A)** Structural model of FhaC with the positions of the residues mutated to Cys for MTSL labeling. Cys503 in the extracellular L7 loop was chosen as a reference point in FhaC (Guérin et al., 2015). **(B,C)** Dipolar evolution function for FhaC^195R1+503R1^ (top), and distance distributions obtained by Tikhonov regularization of the dipolar evolution functions (bottom) for FhaC^33R1+503R1^ (orange), FhaC^187R1+503R1^ (blue), and FhaC^195R1+503R1^ (green) in bOG **(B)** and in proteoliposomes prepared with *E. coli* polar lipids (PL, **(C)**). The black lines in the upper panels correspond to the fitting of the experimental PELDOR traces. The inset in **(B)** represents the first 2.5 µs of the dipolar evolution function, for better comparison with the graph shown in **(C)**. The red dashed lines denoted S1 and S2 show the slopes of the first parts of the curves representing the dipolar evolution functions in bOG and in PL. Note that the longest distances measured depend on the dipolar evolution time *t* (see Supplementary Figure S8).

We introduced a Cys residue at position 503 in the extracellular L7 loop, using it as a fixed reference position in the protein, as in previous PELDOR experiments (Guérin et al., 2014; Guérin et al., 2015), and combined it with another Cys either at position 195 in the b5-b6 hairpin of the POTRA2 domain, position 187 in the b5 strand, or position 33 in the linker (Figure 4A). We then tagged the Cys residues with MTSL, yielding FhaC^33R1+503R1^, FhaC^187R1+503R1^, and FhaC^195R1+503R1^. We recorded CW-EPR spectra of the three proteins in β-octylglucoside (bOG) detergent micelles and in proteoliposomes. The spectra of FhaC^187R1+503R1^ and FhaC^195R1+503R1^ in proteoliposomes exhibited signal broadening compared with the proteins in bOG (shown for FhaC^195R1+503R1^ in Supplementary Figure S6), as previously observed with the singly labeled variants (Guérin et al., 2014; Guérin et al., 2015). The spectral components corresponding to the largest values around 7 mT indicate very slow motions for the spin probe, suggesting interactions with the hydrophobic region of the bilayer. As the spectra of FhaC^33R1+503R1^ were identical in bOG micelles and in liposomes (data not shown), the spectral changes for the other two proteins most likely reflect proximity of the spin probe at positions 187 and 195 to the lipid bilayer.

To explore these conformational changes further with explicit distance measurements, we performed PELDOR experiments. In bOG micelles, for FhaC^195R1+503R1^ and FhaC^187R1+503R1^, the main populated states correspond to distance distributions between the two spin probes that are consistent with distances calculated using MTSL rotamer libraries attached to the corresponding residues of the FhaC crystal structure (Figure 4B; Supplementary Figure S7) (Jeschke, 2013; Jeschke, 2020). For FhaC^33R1+503R1^, a broad distance distribution was observed, with contributions centered at 4.2 and 4.6 nm as predicted by rotamer libraries. For FhaC^195R1+503R1^ and FhaC^187R1+503R1^ in proteoliposomes, the main populated states correspond to long distances of 5–6 nm between the two spin probes (Figure 4C). Note that these distances are shorter than those calculated, since the lipid environment limited the dipolar evolution times that could be applied in PELDOR experiments (Supplementary Figure S8). In addition to the expected long inter-spin distance, shorter distance distributions centered at 2.5 and 3.5 nm were observed for FhaC^195R1+503R1^ (Figure 4C) that can be attributed to conformers with the two spin probes closer to one another than in the resting conformation. Similarly, additional peaks corresponding to shorter-than-expected distances were observed in the distance distributions for FhaC^187R1+503R1^ (Figure 4C). These results strongly support the idea that the b5-b6 hairpin of the POTRA2 domain moves towards the extracellular surface of FhaC in some conformers. For FhaC^33R1+503R1^, distances both shorter and longer than expected were obtained, indicating that in addition to moving away from the membrane when H1 exits the pore (Guérin et al., 2014), the linker also moves toward the surface in specific conformers.

We determined whether the interaction of FhaC with its substrate FhaB affects these conformational changes. The N-terminal region of FhaB binds the POTRA domains of FhaC in the course of secretion; *in vitro*, the proteins interact, but transport does not proceed (Delattre et al., 2011; Guérin et al., 2014). Addition of a minimal substrate, Fha30^N66I^, to FhaC^195R1+503R1^ in proteoliposomes did not affect the distance distribution (Supplementary Figure S9), corroborating the observation that conformational changes are intrinsic to FhaC. We also tested the effect of a point mutation, Asp^492^Arg, that disrupts a conserved salt bridge between L6 and the inner barrel wall, which decreases the secretion activity of FhaC by about 50% and affects the conformation of the protein (Guérin et al., 2015). PELDOR experiments on FhaC^195R1+503R1^ harboring the Asp^492^Arg substitution showed an increased proportion of species characterized by short inter-spin distances (Supplementary Figure S9), suggesting that dissociation of L6 from the barrel facilitates conformational changes of the POTRA2 domain.

Notably, we did not obtain indications for alternative conformers of FhaC reconstituted into nanodiscs with EPR or NMR spectroscopy. The CW-EPR spectra of FhaC^195R1+503R1^ were similar to those in bOG (Supplementary Figure S6C), PELDOR experiments yielded only a long distance between the spin labels (Supplementary Figure S10), and PRE experiments on FhaC^195R1^ showed attenuation of NMR signals only within the POTRA2 domain (Supplementary Figure S5). These findings suggest that the constrained nanodisc environment limits the conformational space accessible to FhaC and hinders the larger-scale conformational changes observed in proteoliposomes.

Taken together, our data show that the POTRA2 domain can spontaneously undergo large conformational changes that bring its b5-b6 hairpin closer to the membrane and the extracellular side, irrespective of the presence of its substrate. The H1-POTRA1 linker can also move towards the cell surface.

### Structural Analysis of FhaC by Native MS

**FIGURE 5:**
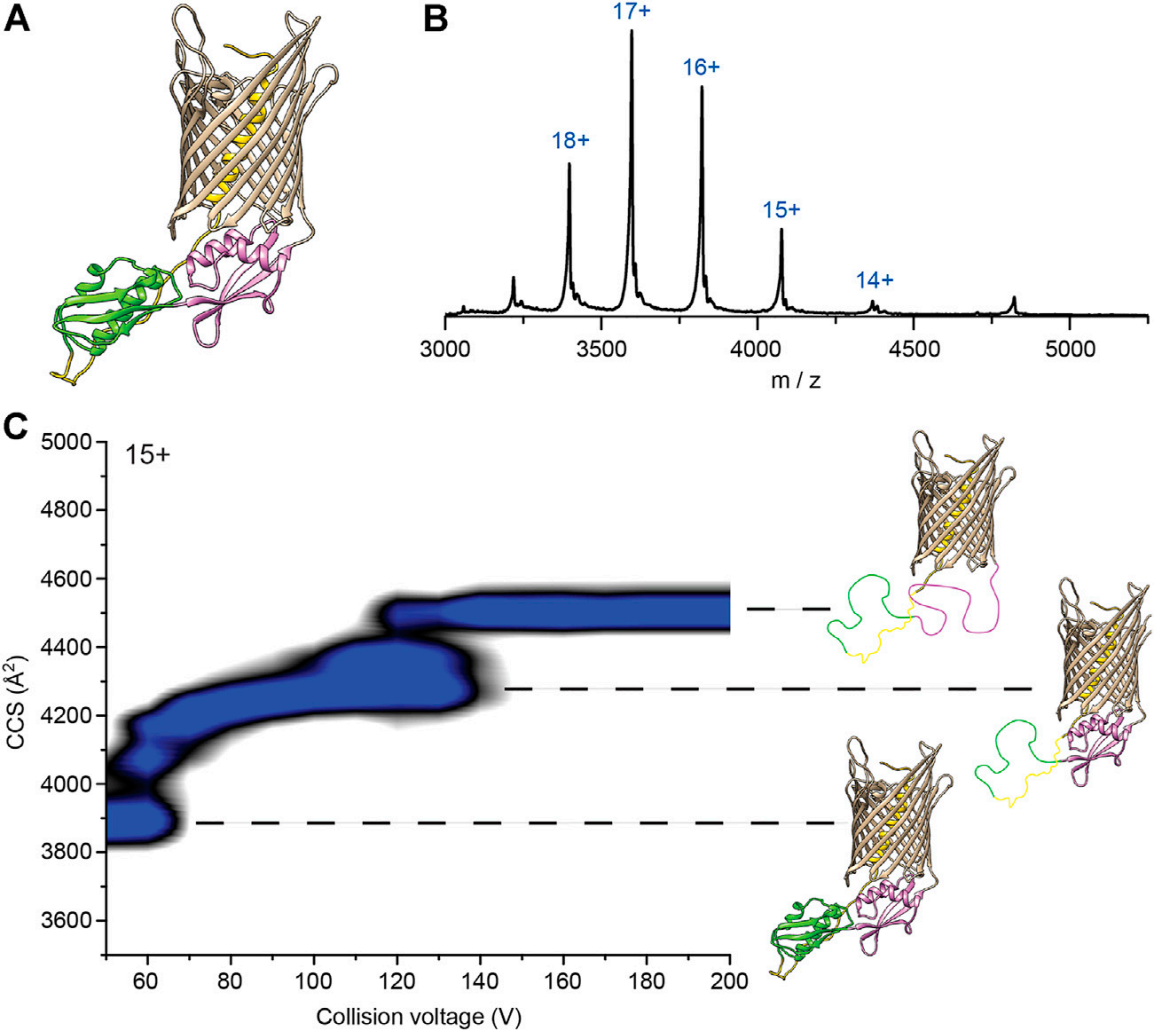
Native mass spectrometry analysis of WT FhaC. **(A)** Model of FhaC with H1 and the linker in yellow, and the POTRA1 and POTRA2 domains in green and pink, respectively. **(B)** Mass spectrum of WT FhaC released from its bOG micelle. The ionization state of the protein is indicated above each peak. **(C)** CIU experiments show two dominant transitions that are likely linked to unfolding of the two POTRA domains (see text). The order in which they unfold is unknown and is depicted here in an arbitrary manner.

Our data imply that the POTRA2 domain undergoes some breaking up in the secretion cycle. We thus investigated its lability by using structural MS-based approaches (Figure 5). Native MS analysis of FhaC revealed a monomer that could be stripped of bOG at 60 V, a relatively low collision energy (CE), and that displayed a narrow charge state distribution between 14+ and 19+ indicative of a folded protein in a single conformation (Figures 5A,B, Supplementary Figure S11).

We used collision-induced unfolding (CIU) (Tian et al., 2015) to characterize the stability and the organization of the FhaC domains. FhaC displayed two transitions at 60 and 120 V as shown by the increases of collision cross section (CCS) values (Figure 5C). As the number of transitions in the gas phase can generally be related to the number of domains of a protein (Zhong et al., 2014), and extra-membrane domains are more likely to experience early unfolding than domains embedded in detergent or lipids due to collisional cooling (Barrera et al., 2009), those transitions might be caused by unfolding of the POTRA domains and/or ejection and unfolding of H1. Control CIU experiments with other *B. pertussis* OMPs with small soluble domains inside their β barrels, the TonB-dependent transporter BfrG and the translocator domain of an autotransporter, SphB1-αβ, showed a single unfolding transition at low voltage, which likely corresponds to unfolding of their soluble domains (Supplementary Figure S12). Thus, the β barrels of these three proteins likely remain structurally intact at high activation conditions, most likely due to strong hydrogen bonding networks.

To further investigate whether the CIU transitions observed for FhaC stem from unfolding of the POTRA domains or ejection of the H1 helix, we studied the CIU pathway of FhaC^C4+C391^ in which H1 is locked inside the barrel by an S-S bond and thus cannot move out (Guérin et al., 2014). FhaC^C4+C391^ exhibited the same transitions as wt FhaC, although the second unfolding event was delayed by 30 V and the overall CCS value was increased by 50 Å^2^ (Supplementary Figure S13). As CIU is unlikely to break S-S bonds (Tian et al., 2015), comparison of these unfolding pathways suggests that the two transitions correspond to successive unfolding of the POTRA domains, with the barrel remaining intact in those conditions. H1 stays inside the barrel or its unfolding barely registers in the CCS values. The delay of the second unfolding transition for FhaC^C4+C391^ suggests that locking H1 in the barrel stabilizes one of the POTRA domains, although from the data we cannot discern which one.

**FIGURE 6:**
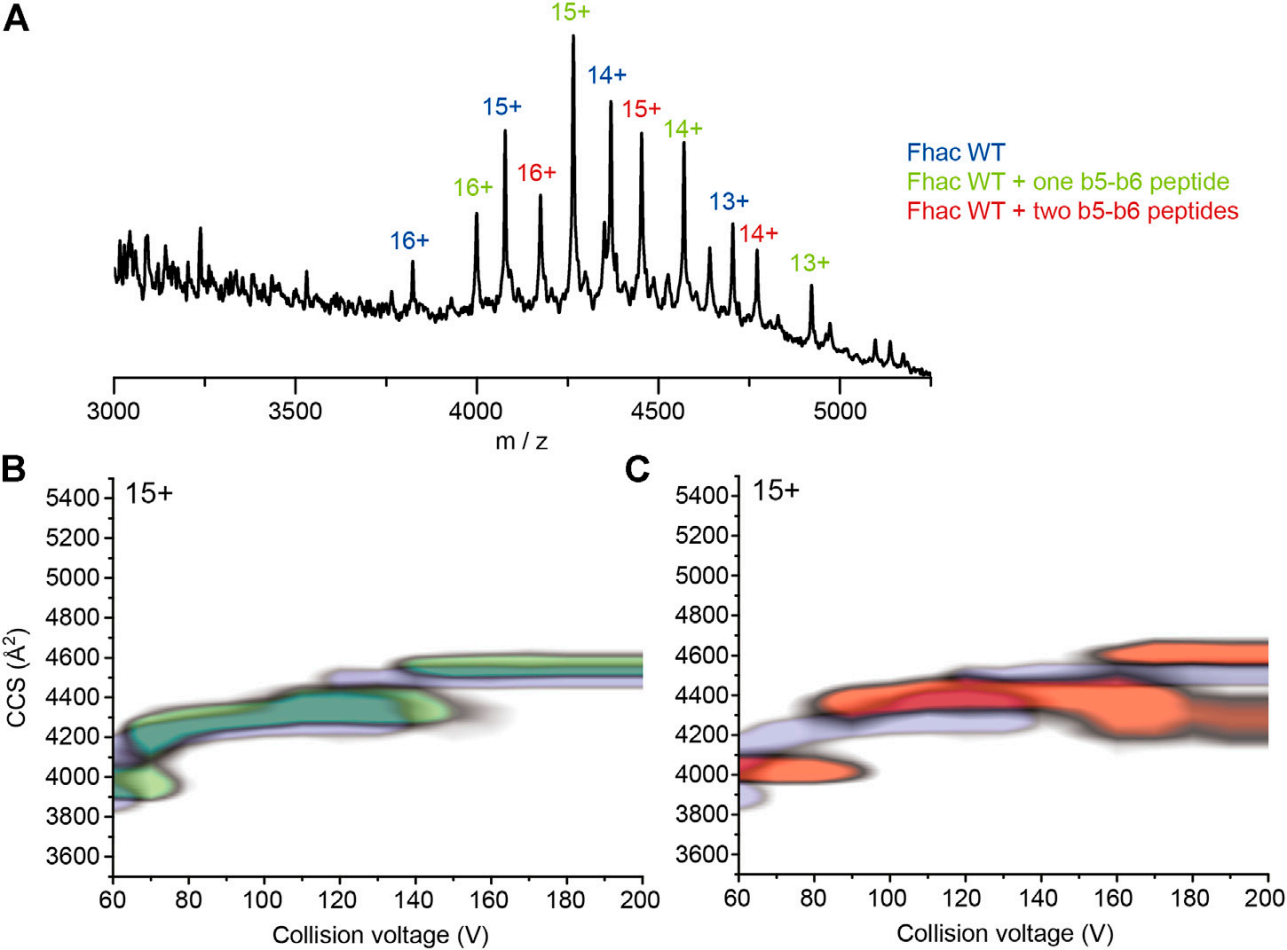
Binding of synthetic peptides to FhaC. **(A)** Mass spectrum of FhaC alone or incubated with the b5-b6 peptide in conditions of high activation energy (CE = 150 V). The ionization state of the protein is indicated above each peak. The species indicated in blue correspond to FhaC alone, those in green correspond to FhaC with one peptide bound, and those in red to FhaC with two peptides bound. **(B)** Overlay of the CIU plots of FhaC with (green) and without (blue) one b5-b6 peptide bound. Increased CCS values are observed both under native conditions (low CE) and conditions in which the POTRA domains are most likely unfolded (high CE). **(C)** Comparison of the CIU plots of FhaC alone (blue) and FhaC with two b5-b6 peptides bound (orange), which shows an additional CCS increase compared to FhaC with a single peptide bound.

We next tested the possibility that portions of the POTRA2 domain might bind to the β barrel upon partial disruption of the barrel seam. Using native MS, we assessed the binding of synthetic peptides that correspond to various periplasmic portions of FhaC, including b5-b6, b4+L (*i.e.*, b4 followed by the b4-H3 linker) and L + H4 (*i.e.*, the H3-H4 linker followed by H4) of the POTRA2 domain, b2-b3 of the POTRA1 domain, Lk, a non-structured peptide from the linker region between H1 and the POTRA1 domain, and the N-terminal β hairpin of the FhaB transport substrate, Fha-NT (Figure 6, Supplementary Figure S14). The same experiments were performed with SphB1-αβ to correct for non-specific binding, which might occur in native MS experiments due to artifacts induced by interaction with the detergent during the electrospray process (Landreh et al., 2016). Fha-NT, b4+L and b5-b6 exhibited binding to FhaC, with b5-b6 binding at the highest level and in two copies, but markedly less to the FhaC^C4+C391^ variant (Figure 6A, Supplementary Figure S15). In contrast, the peptides containing the sequences of b2-b3 of the POTRA1 domain, H4 or the H1-POTRA1 linker did not bind.

We assessed structural changes induced by peptide binding using native ion-mobility MS. At low CE (*i.e.*, no activation), all three peptides increased the CCS of the compact state of FhaC by rather small increments of 91–92 Å^2^ (Figure 6B,C, Supplementary Figure S16). However, upon increasing the activation conditions, Fha-NT and b4+L no longer increased the CCS of FhaC, compared to the unbound protein. In contrast, the b5-b6 peptide caused an increase in CCS values both at low and high collisional activation, suggesting that a structural change was induced upon peptide binding and that the peptide was bound to a region that remains folded in these conditions (Figures 6B,C, Supplementary Table S17). As our CIU studies indicate that the POTRA domains likely unfold at high CE, the effect of b5-b6 on the CCS might thus stem from peptide binding to the β barrel. The same experiment with FhaC^C4+C391^ showed a lower level of peptide binding, which nevertheless caused a similar increase of CCS at both low and high energies, like with wt FhaC (Supplementary Figure S16 and Supplementary Table S17). This is consistent with the model that the peptide corresponding to the b5-b6 hairpin of the POTRA2 domain interacts with the β barrel, and that this interaction is facilitated by the ejection of H1.

### *In vivo* Effects of Freezing the Conformation of the POTRA2 Domain

**FIGURE 7:**
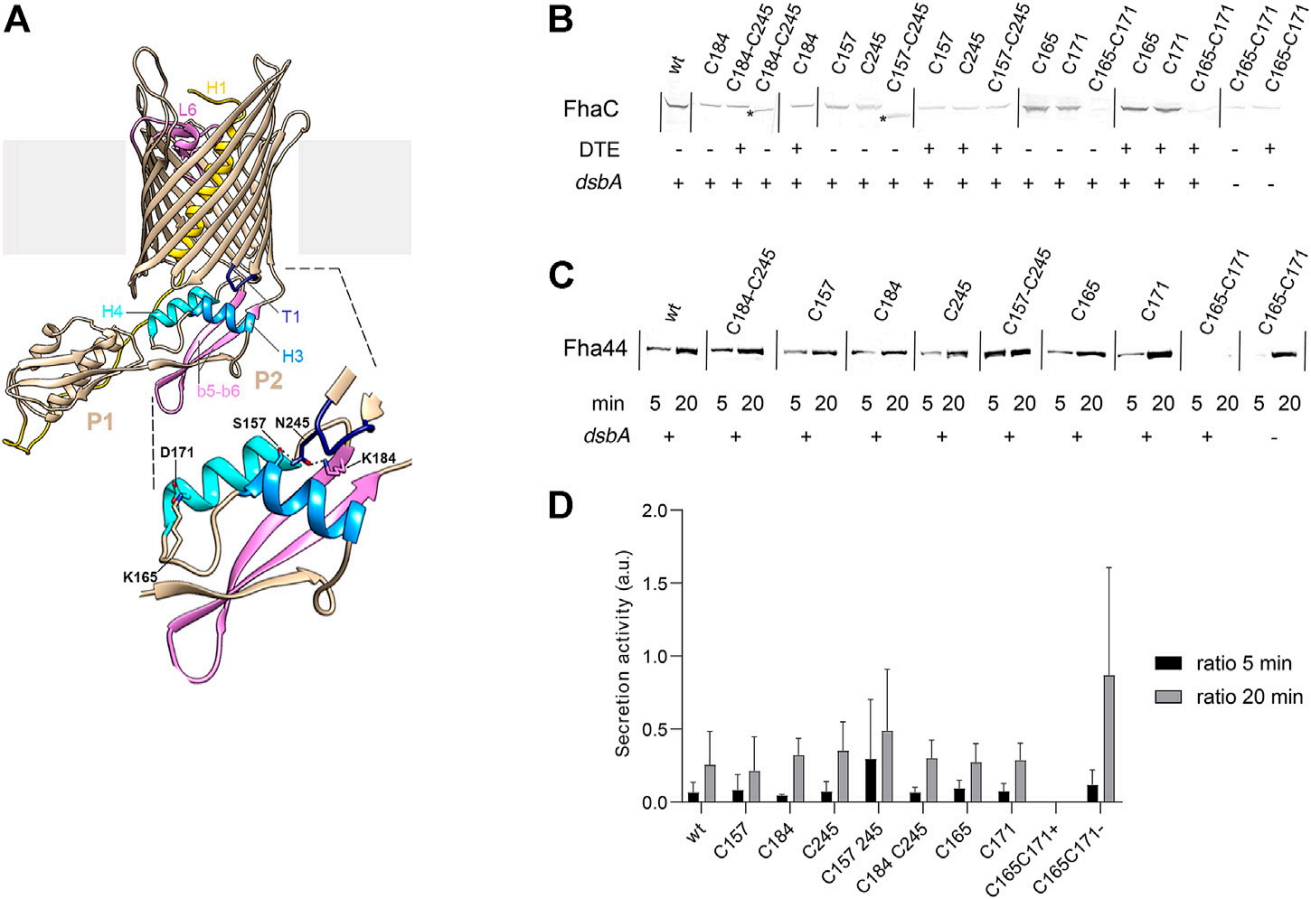
Effects of engineered S-S bonds on FhaC activity. **(A)** Structural model of FhaC with a zoom of the POTRA2 domain shown below. **(B)** Residues involved in a salt bridge (Lys^165^-Asp^171^) or H bonds (Lys^184^-Asn^245^; Ser^157^-Asn^245^) in the resting conformation of FhaC were replaced as indicated (C=Cys). Immunoblots were performed on membrane extracts with anti-FhaC antibodies. The asterisks indicate oxidized species of FhaC detected in the absence of the reducing agent DTE in the sample buffer. **(C)** The secretion activity of the FhaC variants was determined using a model substrate, Fha44-His, affinity precipitated from supernatants 5 and 20 min after induction. Immunoblots were developed with an anti-6His tag monoclonal antibody. **(D)** Quantification of Fha44 found in culture supernatants after 5 and 20 min, normalized by the expression levels of the respective FhaC mutants. The means and standard deviations of the means are shown (n = 3 or 4). Activity of FhaC^C165+C171^ could only be detected in the *dsbA* KO strain (denoted C165-C171 (-)), not in its wild type parent (denoted C165-C171 (+)).

The alternative FhaC conformers suggested by our data imply a re-orientation of the POTRA2 domain relative to the barrel. To probe this *in vivo*, we searched for specific H bond- or salt bridge-mediated interactions present in the resting conformation and replaced them with S-S bonds to limit motions of the corresponding regions. We replaced residues involved in interactions between the POTRA2 domain and the barrel (Asn^245^-Ser^157^ and Asn^245^-Lys^184^) and in a barrel-distal region of the POTRA2 domain (Asp^165^-Lys^171^) with Cys, and we determined the effects of these mutations on secretion activity (Figure 7A–D). S-S bond formation had different effects depending on the sites involved. The Cys^157^-Cys^245^ and Cys^184^-Cys^245^ S-S bonds were compatible with FhaC secretion activity (Figure 7), whereas the engineered Cys^165^ + Cys^171^ substitutions abolished it in a *dsbA* ^*+*^ background, but not in a *dsbA* ^*-*^ background. Therefore, FhaC activity requires flexibility of the H4 helix N terminus, most likely because this region moves in the course of secretion. In contrast, transport activity does not require disruption of the interface between the first periplasmic turn of the barrel and barrel-proximal elements of the POTRA2 domain.

## Discussion

As Omp85 proteins are thought to perform their functions in the absence of an energy source in the periplasm, their postulated conformational cycling must involve low energy barriers between conformers, as reported for BamA (Xiao et al., 2021). Here, we obtained evidence for large conformational changes of FhaC that involve portions of the POTRA2 domain moving toward the extracellular side of the protein. Conformational changes of FhaC occur independently of the presence of the substrate, indicating that such dynamics is an intrinsic structural feature of the protein, with implications for its function. As indicated by NMR relaxation dispersion data, the conformational states appear to be in slow equilibrium, i. e., on time scales longer than microseconds, as in BamA (Hartmann et al., 2018).

All structural elements of TpsB transporters are connected with one another, structurally and functionally, and their motions appear to be coupled (Guérin et al., 2014; Guérin et al., 2015; Maier et al., 2015; Guérin et al., 2020). In the resting conformation, H1 and L6 interact with the barrel wall, H1 with L1, the H1-POTRA1 linker with the POTRA domains, and the POTRA2 domain with the periplasmic side of the barrel. In the secretion process, L6 breaks its connection with the barrel wall, H1 moves towards the periplasm, and part of the B1-B16 seam unzips (Guérin et al., 2014; Guérin et al., 2015; Guérin et al., 2020). Of note, it has been shown that partial opening of the B1-B16 seam is required for two-partner secretion (Guérin et al., 2020).

In this work we obtained evidence for further conformational changes involving the POTRA2 domain. Thus, EPR, NMR, and S-S cross-linking data revealed that parts of the POTRA2 domain approach the barrel seam and the extracellular side of FhaC in specific conformers, and structural MS experiments indicated the binding of a synthetic peptide corresponding to the last portion of POTRA2 to the β barrel. We propose that this peptide binds to the B1 strand of the partially unzipped barrel seam based on the following reasoning. The number of unfolding transitions we observed in CIU experiments in FhaC and control β barrel proteins corresponds to the number of their soluble domains, which are generally more likely to unfold in these experiments than lipid- or detergent-embedded β barrels (Barrera et al., 2009). At high collision energy, when the POTRA domains of FhaC are most likely unfolded, binding of control peptides to FhaC was lost; binding of the b5-b6 peptide was however observed to persist, as well as to increase the collision cross section of FhaC (Figure 6B,C), suggesting binding to the β barrel itself. Alternative interpretations of the CIU data, in particular that the b5-b6 peptide binds to a POTRA domain by β augmentation and increases its stability, cannot be ruled out. However, it appears unlikely that this binding could persist at very high collision energy, when the soluble POTRA domains are most probably unfolded.

Our EPR, NMR, and S-S crosslinking results indicate that the last portion of the POTRA2 domain approaches the extracellular side of the protein spontaneously. Since the b5-b6 peptide immediately precedes the B1 strand of the barrel, interaction of these regions in a partially opened β−barrel seam is likely facilitated in the native protein compared to the situation in MS experiments. The b5-b6 hairpin is amphipathic and exhibits suitable charge partitioning for β augmentation of the B1 strand. Such an interaction would be in line with the molecular mechanisms of BamA and Sam50, in which the unzipped B1 strand templates folding of client proteins by β augmentation (Höhr et al., 2018; Doyle and Bernstein, 2019; Tomasek et al., 2020; Wu et al., 2021).

We thus propose that the spontaneous conformational changes of the POTRA2 domain initiate secretion as follows. Exit of H1 facilitates partial unzipping between B1 and B16, coupled with partial unfolding of the POTRA2 domain and a swing motion of its b5-b6 hairpin - and possibly also H4 - towards the barrel seam. Partial disruption of the barrel seam enables binding of part of the b6 strand of the flipped POTRA2 hairpin to barrel strand B1. Since the groove between H4 and b5 is the substrate binding site (Delattre et al., 2011), this conformational change brings the bound substrate into the channel. The part of the POTRA2 domain that moves into the barrel likely remains in that position during secretion. The observation that portions of the H1-POTRA1 linker can also approach the extracellular loops likely reflects futile conformational changes involving the linker bound to POTRA2 being hoisted into the channel. Notably, evidence for the C-terminal portion of the linker reaching the cell surface was obtained previously (Guédin et al., 2000).

Although the next secretion steps are speculative, we propose the following model based on earlier work. The substrate downstream of the segment bound to b5 forms a hairpin inside the channel, and the entropic cost of confining a portion of the unfolded polypeptide facilitates its diffusion toward the surface (Halladin et al., 2021). We have identified specific interactions between the TPS domain and the extracellular β sheet formed by barrel strands B5-B8 that might template folding of the substrate into a nascent β helix (Baud et al., 2014). Progressive threading of the polypeptide across the channel and its folding at the surface eventually lead to the formation of a stable β helix nucleus (Alsteens et al., 2013), which prevents backtracking of the protein chain, adding directionality to its stochastic motion as proposed by the “Brownian ratchet” mechanism (Peterson et al., 2010). The molecular interactions between FhaC and FhaB are similar to those proposed in an earlier model (Nash and Cotter, 2019), in which the TPS domain first binds to the POTRA2 domain and then progressively folds using the extracellular β sheet of FhaC as a template, with formation of a hairpin for translocation of the rest of the protein. The major differences between the two models are the conformation and the position of the POTRA2 domain during secretion.

Using an *in vitro* translocation assay, it has been shown that FhaC mediates protein secretion without the need for cofactors or energy (Fan et al., 2012), and this work indicates that this is accomplished by utilizing intrinsic protein dynamics. Although the mechanism that we describe is different from those observed in other Omp85 proteins, divergent functional evolution has necessarily led to specific mechanistic adaptations. Intriguingly, we showed that, in contrast to BamA (Iadanza et al., 2020), FhaC cannot undergo conformational changes in nanodiscs, which appear to constitute a more constrained environment than proteoliposomes. Whether this difference is linked to the ability of BamA to distort the lipid bilayer, or to other distinctive features of the Bam complex, remains to be determined. It is also possible that the lack of curvature of the membrane in nanodiscs accounts for the absence of conformational heterogeneity of FhaC in this environment. An effect of mismatched lipid bilayer thickness on FhaC conformational dynamics in nanodiscs is less likely, since FhaC displayed conformational heterogeneity in proteoliposomes made of the same mixtures of pure lipids as those used in nanodiscs (Supplementary Figure S6).

According to our model, the POTRA2 domain partially breaks up during secretion and must thus reassemble after secretion is completed. This may be mediated by interactions between the periplasmic turn T1 of the barrel and barrel-proximal residues of the POTRA2 domain. The observation that replacement of these interactions by S-S bonds is compatible with FhaC activity indicates that they represent fixed points in the protein. By anchoring the POTRA2 domain to the barrel, these interactions may ensure that FhaC can regain its resting conformation after secretion, which is necessary to limit outer membrane permeability. Consistent with this hypothesis, disrupting the conformation of the periplasmic turn T1 yielded transient, very large channels as detected in electrophysiology experiments (Méli et al., 2006). Stabilizing the resting conformation of the transporter may also account for the importance of the interaction between L6 and the inner barrel wall (Delattre et al., 2010).

In summary, we propose a novel mechanism for initiation of protein transport in TPS systems based on large-scale, spontaneous conformational dynamics of the TpsB partner. Our model integrates and explains currently available data from several complementary *in vitro* and *in vivo* approaches and establishes mechanistic links between TpsB transporters and Omp85 insertases.

## Data Availability

The datasets presented in this study can be found in online repositories. The raw EPR, native mass spectrometry and NMR data have been deposited in a Zenodo repository (DOI 10.5281/zenodo.5831843, https://zenodo.org/record/5831843). NMR assignments for isoleucine δ1 methyl groups of FhaC in nanodiscs, where all 15 isoleucine signals are present and resolved, have been deposited in the Biological Magnetic Resonance Data Bank (BMRB) under accession number 51444 (DOI 10.13018/BMR51444, https://bmrb.io/data_library/summary/?bmrbId=51444).
