# Supplementary material for: Large-Scale Conformational Changes of FhaC Provide Insights Into the Two-Partner Secretion Mechanism
Source: Front Mol Biosci. 2022 Jul 22;9:950871. doi: 10.3389/fmolb.2022.950871 (PMC9355242; doi:10.3389/fmolb.2022.950871)
Supplement: Supplementary file 1 [file DataSheet1.PDF]

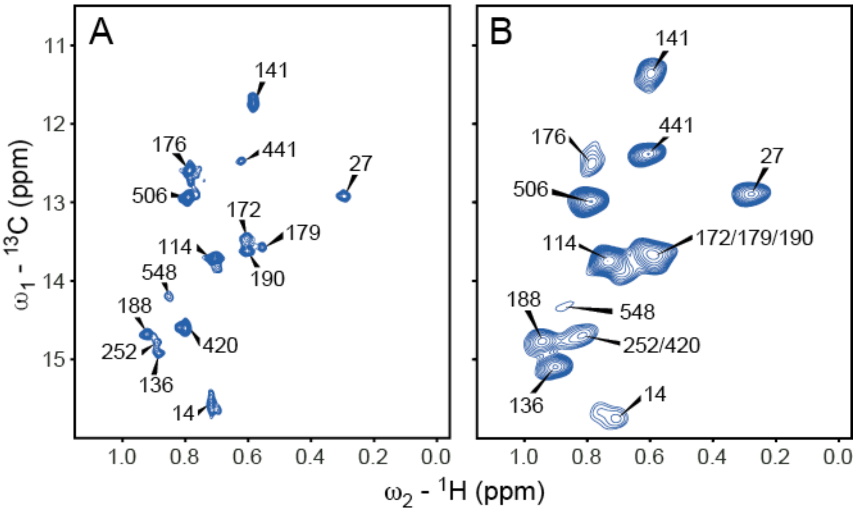

Figure S1. NMR analyses of Ile δ_1 methyl labeled FhaC in lipid bilayers. (A) Methyl region of a solution-state heteronuclear multiple quantum coherence (HMQC) ^{13}C-^{1}H correlation spectrum of u-(^{2}H, ^{15}N), Ile-δ_1($^{13}CH_3$)-labeled FhaC^{195R1} in ^{2}H-MSP1D1 nanodiscs prepared from deuterated (d_{54}-) DMPC and DMPG (2:1) lipids, recorded on a 900 MHz spectrometer. The MTSL tag on residue 195 was reduced with ascorbic acid; peak positions are identical to those of wt FhaC in nanodiscs. (B) Same region of a scalar coupling-based solid-state J-HSQC ^{13}C-^{1}H correlation spectrum of wt FhaC (same isotope labeling as in (A)) in d_{54}-DMPC/DMPG liposomes, recorded on an 800 MHz spectrometer at 50 kHz MAS.

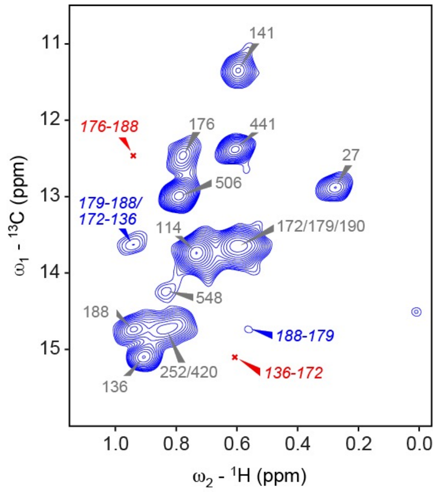

Figure S2. NMR analysis of through-space contacts between Ile δ_1 methyl groups in FhaC in liposomes. 2D hChH correlation spectrum with 6.4 ms RFDR (1) ^{1}H-^{1}H mixing of FhaC u-(^{2}H, ^{15}N), Ile-δ_1($^{13}CH_3$) in deuterated (d_{54}-) DMPC/ DMPG liposomes, recorded on an 800 MHz NMR spectrometer at 50 kHz MAS, to visualize through-space correlations between Ile δ_1 methyl groups close in space. Among expected inter-residue cross-peaks (^{1}H-^{1}H distance below 6 Å), peaks present in the spectrum are indicated in blue, those which are absent in red.

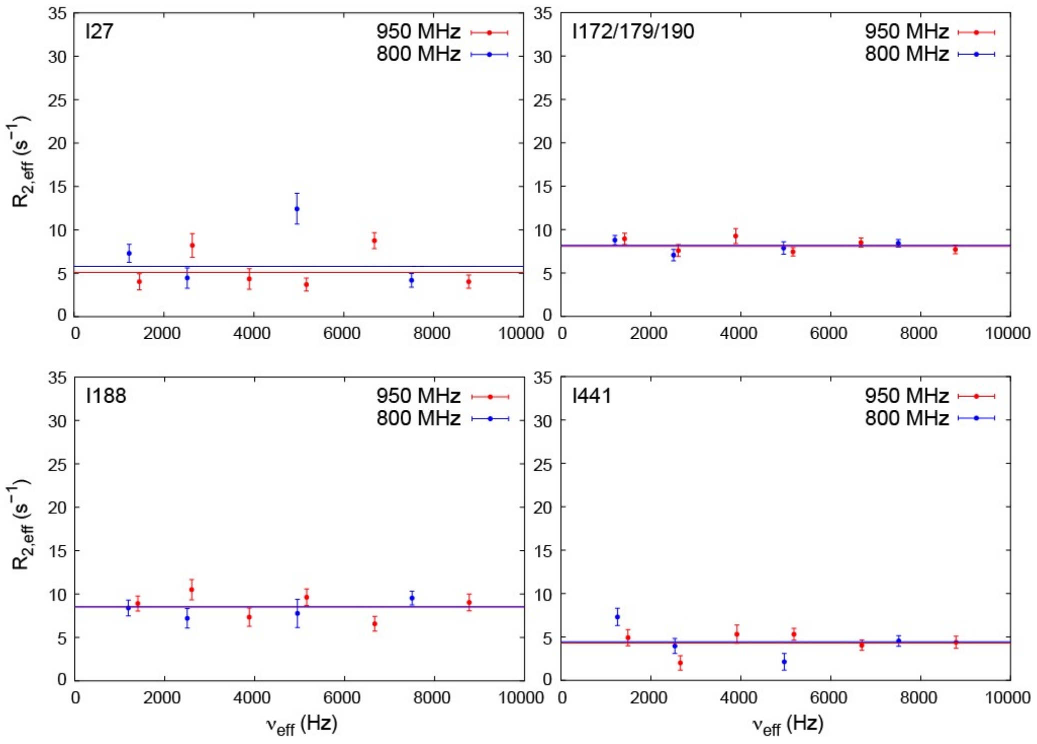

Figure S3. NMR relaxation dispersion experiments to measure μs time scale exchange dynamics in FhaC. Effective ^{13}C transverse relaxation rates $R_{2,eff}$ extracted from solid-state NMR $R_{1\rho}$ relaxation dispersion experiments (2, 3) on selected Ile-δ_1 methyl groups of u-(^2H, ^{15}N), Ile-δ_1($^{13}CH_3$)-labeled wt FhaC, recorded on 800 (blue) and 950 MHz (red) spectrometers at 50 kHz MAS frequency and 17°C sample temperature. Horizontal lines are best fits to the data using a model of no exchange (i.e. constant $R_{2,eff}$ values for varying applied B_1 radiofrequency fields and thus varying effective fields ν_{eff}). Models assuming exchange do not fit the data significantly better according to F test statistics in any of the Ile-δ_1($^{13}CH_3$) groups of FhaC. Notably, data from residue Ile^{548} in strand β16, at the barrel junction with strand β1, could not be reliably analyzed due to low signal-to-noise.

FhaC^{187R1}		FhaC^{195R1}		FhaC^{220R1}	
Ile residue	Avg. dist. to paramag. center (Å)	Ile residue	Avg. dist. to paramag. center (Å)	Ile residue	Avg. dist. to paramag. center (Å)
176	8.3	114	16.6	14	7.1
188	11.1	136	18.2	252	11.7
179	13.0	190	21.0	548	16.5
172	13.5	172	22.1	27	17.5
190	13.6	141	23.2	441	18.6
141	16.3	188	25.1	506	25.3
136	18.6	179	27.8	420	32.0
114	27.3	176	28.4	179	32.1
27	28.5	27	50.8	176	34.1
420	30.2	252	54.6	188	35.4
252	34.2	420	59.5	141	38.4
441	40.7	441	60.5	172	40.4
548	40.7	14	63.2	136	42.0
14	42.6	548	69.1	190	43.5
506	52.4	506	74.9	114	56.6

Table S4. Estimated Ile Cδ_1 – MTSL distances in the crystal structure conformations of FhaC^{187R1}, FhaC^{195R1}, and FhaC^{220R1}. Shown are distances (in Å) between Ile Cδ_1 nuclei and the estimated average positions of the paramagnetic centers in FhaC with MTSL spin labels on residues 187 (FhaC^{187R1}, left), 195 (FhaC^{195R1}, center), and 220 (FhaC^{220R1}, right). Ensembles of 200 MTSL conformations compatible with labeling on these residues were calculated using the mtsslSuite web server or PyMOL plugin (4, 5); http://www.mtsslsuite.isb.ukbonn.de/) and the FhaC crystal structure (PDB 4QKY). Distances from the mean positions of the paramagnetic center to Ile Cδ_1 nuclei were calculated using PyMOL (The PyMOL Molecular Graphics System. Schrödinger, LLC). Horizontal lines in the table indicate the distance from the paramagnetic center up to which attenuation effects on the NMR resonances of the corresponding Ile residues are expected (~ 30 Å) if FhaC assumes a conformation as in the crystal structure.

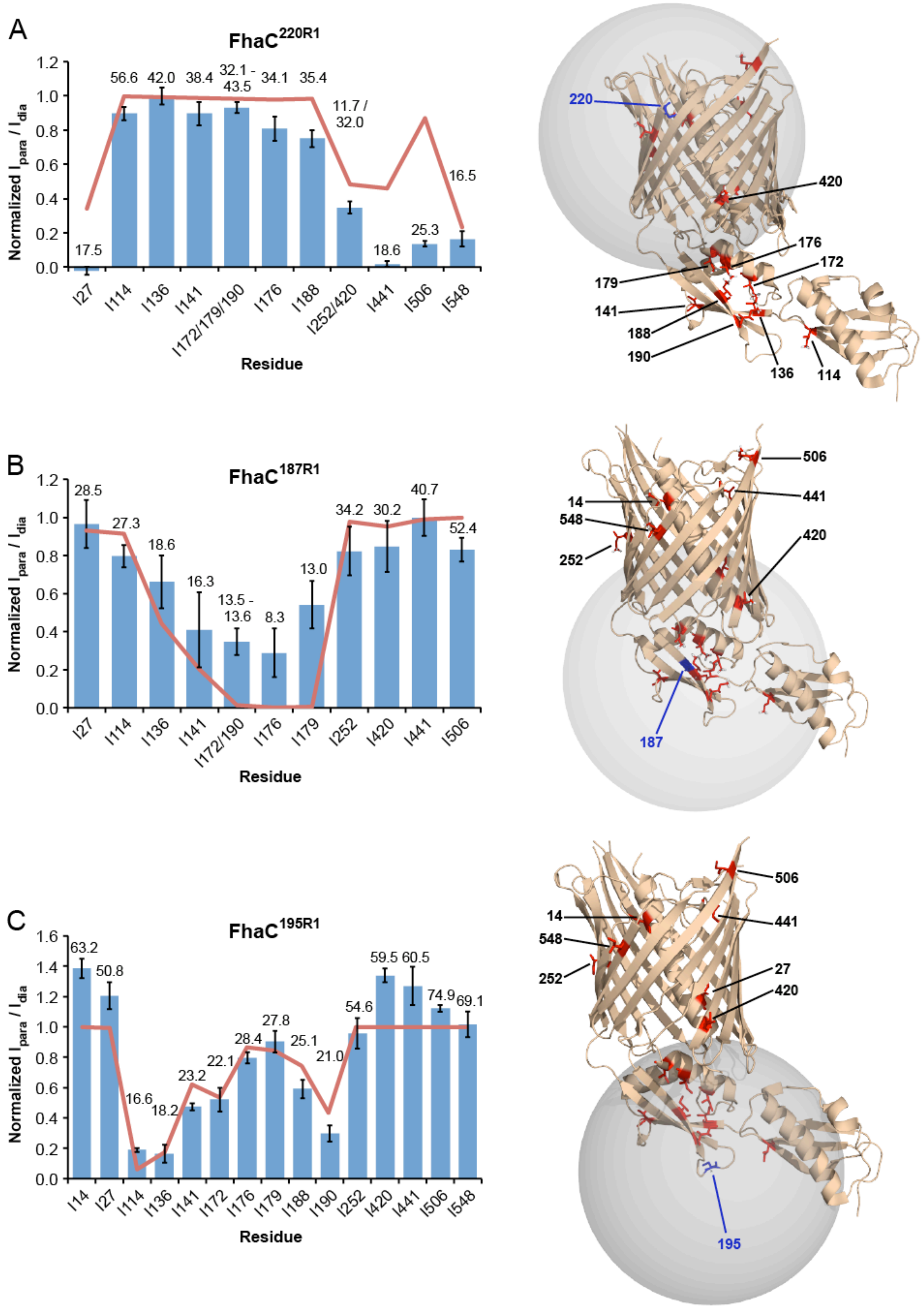

Figure S5. NMR paramagnetic relaxation enhancement experiments on FhaC. Shown are experimental (blue bars) and calculated (red lines) PREs (left) and the approximate sphere

of influence (30 Å radius) around the paramagnetic center introduced in the FhaC crystal structure (PDB 4QKY, right) for FhaC^{220R1} (A, same data as in Figure 3 in the main text), FhaC^{187R1} (B), and FhaC^{195R1} (C). Data for FhaC^{220R1} and FhaC^{187R1} were measured by solid-state NMR on the proteins in liposomes (dipolar hCH correlation spectra at 800 MHz ^{1}H Larmor frequency, 17°C sample temperature, 50 kHz MAS); data for FhaC^{195R1} were obtained by solution-state NMR on the protein reconstituted in nanodiscs (^{13}C-^{1}H SOFAST-HMQC spectra at 900 MHz ^{1}H Larmor frequency, 32°C sample temperature). PREs were calculated as ratios of Ile-δ_1 methyl peak intensities in para- vs. diamagnetic samples (I_{para}/I_{dia}). In case of FhaC^{220R1}, the paramagnetic sample was a separate sample tagged with a diamagnetic MTSL analogue; for FhaC^{187R1} and FhaC^{195R1}, the paramagnetic MTSL sample was reduced by an excess of ascorbic acid to yield a diamagnetic sample (see Methods in the main text).

PREs are normalized to their maximum value for the samples measured by solid-state NMR to correct for different amounts of protein reconstituted into liposomes as well as transferred to solid-state NMR rotors between para- and diamagnetic samples (FhaC^{220R1}), and for sample loss due to removing the sample from the rotor for reduction (FhaC^{187R1}). Values for FhaC^{195R1} are not normalized; values greater than 1 can be explained by incomplete relaxation in the diamagnetic sample due to short recycle delays used to obtain maximum signal per unit time.

For theoretical estimates of PREs, distances between Ile Cδ_1 atoms and the average position of the paramagnetic center modeled onto the respective Cys residue in the FhaC crystal structure were used (shown above the bars; same values as in Table S4). Cys mutations were introduced in the FhaC crystal structure via PyMOL (The PyMOL Molecular Graphics System, Schrödinger, LLC; https://pymol.org/). For modeling the paramagnetic center, 200 MTSL conformations compatible with the respective position in the FhaC crystal structure were generated using the mtsslWizard PyMOL plugin or the mtsslSuite web server (4, 5).

As is customary, for the PRE, the paramagnetic contribution to ^{1}H transverse relaxation (R_2) was calculated using the Solomon-Bloembergen equation, as shown in eq. (5) of reference (6). For solid-state PREs (FhaC^{220R1}, FhaC^{220R1}), the correlation time τ_c of the electron-nuclear spin interaction in this equation was set to 100 ns, the electron transverse and longitudinal relaxation time in a MTSL spin label, since overall rotational tumbling of the protein is absent in the solid state (7). For solution-state PREs (FhaC^{195R1}), τ_c was set to $\tau_r\tau_e$ / ($\tau_r + \tau_e$) with τ_e the electron relaxation time (100 ns) and τ_r the rotational correlation time for a MSP1D1 nanodisc carrying a FhaC molecule, estimated from its total molecular mass of about 253 kDa (1 FhaC + 3 MSP1D1 + 180 lipid molecules) as about 130 ns at 32°C. This yielded a τ_c of 56.5 ns for the electron-nuclear spin interaction of FhaC in nanodiscs.

The PRE itself (I_{para}/I_{dia} intensity ratio) was then calculated using eq. (4) of reference (6) for solid-state data, with t the total ^{1}H-^{13}C transfer time in the hCH pulse sequence (3 ms for 2 cross-polarization transfers of 1.5 ms). For solution-state data, eq. (4) of reference (8) appropriate for HMQC pulse sequences was used, with t set to 8 ms. Diamagnetic R_2 values were estimated from linewidths of isolated peaks in the spectra. Error bars are calculated using standard error propagation based on spectral noise levels.

In the structural models in the right column, FhaC Ile residues are shown as red sticks and the introduced MTSL-bearing Cys is shown in blue. Ile residues outside of the radius of 30 Å around the modeled paramagnetic center (gray sphere), whose NMR signals are not expected to be attenuated, are labeled with their residue number.

In data from FhaC^{220R1} (panel (A)), apart from the effects discussed in the main text, it is apparent that signals due to residues Ile^{27}, Ile^{441}, and Ile^{506} are sizably more attenuated than theoretically expected. While we do not expect quantitative agreement between predicted and measured PREs and have rather based our reasoning on a comparison of attenuation levels between different FhaC residues, these differences can be rationalized by the position of residue 220 in a loop that, in the crystal structure conformation, is folded inward into the barrel. In presence of the bulky MTSL tag, its position may well change (which is not accounted for by mtsslSuite modeling), bringing the tag closer to Ile^{27} at the periplasmic end of H1 on the one hand, or to the extracellular side and thus Ile^{441} and Ile^{506} on the other hand in different conformers. Such loop movement would however not approach the MTSL tag sufficiently towards the POTRA2 domain to account for the observed attenuation in Ile^{188} discussed in the main text.

Note that peaks of residues Ile^{188} and Ile^{548} could not be analyzed in the spectra of FhaC^{187R1} (panel (B)) due to signal overlap and the unknown chemical shift of Ile^{188} in reduced FhaC^{187R1} due to the mutation of the neighboring residue 187. Peaks due to residues Ile^{172}/Ile^{190}, Ile^{176}, and Ile^{179} would be expected to be fully bleached out in FhaC^{187R1}, since they are closer than 15 Å to the paramagnetic center in the crystal structure conformation. Their intensities significantly different from 0 ($p < 0.05$, one-sided Z test) may be rationalized by the b5-b6 hairpin (containing Cys^{187}-MTSL) being removed from POTRA2 and the H4 helix (containing Ile^{172}, Ile^{176}, and Ile^{179}) in some FhaC conformers, in accordance with our model of large-scale dynamics in POTRA2. Conversely, some residues in the barrel appear more attenuated than expected for distances above 30 Å from the MTSL label in the crystal structure, notably Ile^{506} whose I_{para}/I_{dia} ratio is significantly lower than 1 ($p < 0.05$, one-sided Z test), indicating that Cys^{187}-MTSL can approach the barrel exterior.

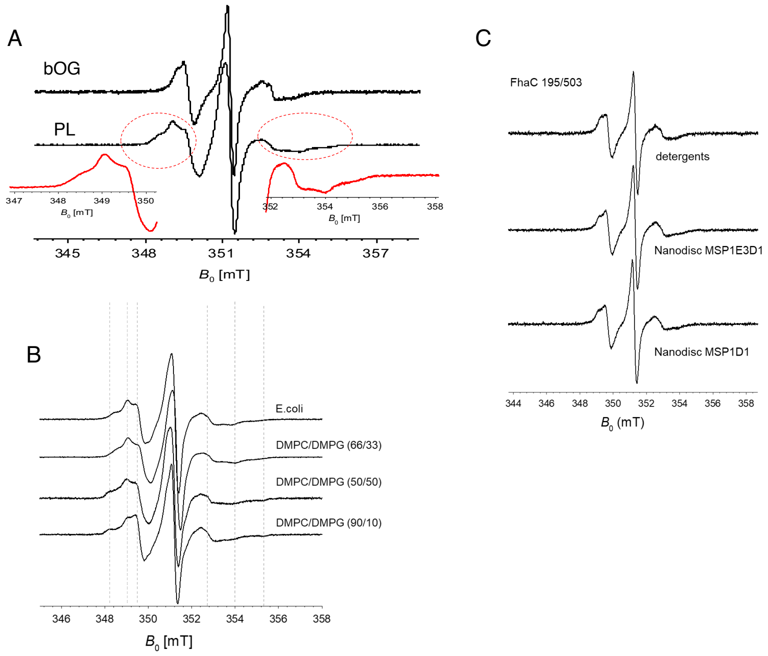

Figure S6. CW EPR spectra for FhaC$^{195R1+503R1}$ in various environments. A. Comparison between bOG micelles and proteoliposomes (PL) made of *E. coli* lipids. The regions circled in red are enlarged below (red traces). B. The comparison of the spectra in PL made of *E. coli* lipids or of mixtures of pure lipids, DMPC and DMPG, shows similar spectral broadening in all cases. C. In contrast, spectra of the protein in nanodiscs are similar to those in bOG, irrespective of the size of the scaffold proteins (small nanodiscs with MSP1D1, larger nanodiscs with MSP1E3D1).

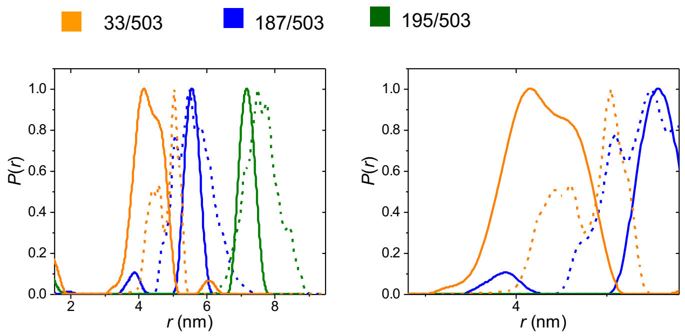

Figure S7. Distance distributions from PELDOR experiments. The distance distributions obtained for FhaC$^{33R1+503R1}$ (orange), FhaC$^{187R1+503R1}$ (blue) and FhaC$^{195R1+503R1}$ (green) in bOG (solid lines) are compared with those predicted using a pre-computed rotamer library of the MTSL spin probe attached to specific residues on the PDB structure of FhaC (dashed lines) (9). In the right panel, a zoom on the 3-5 nm region shows the broad distribution for FhaC$^{33R1+503R1}$.

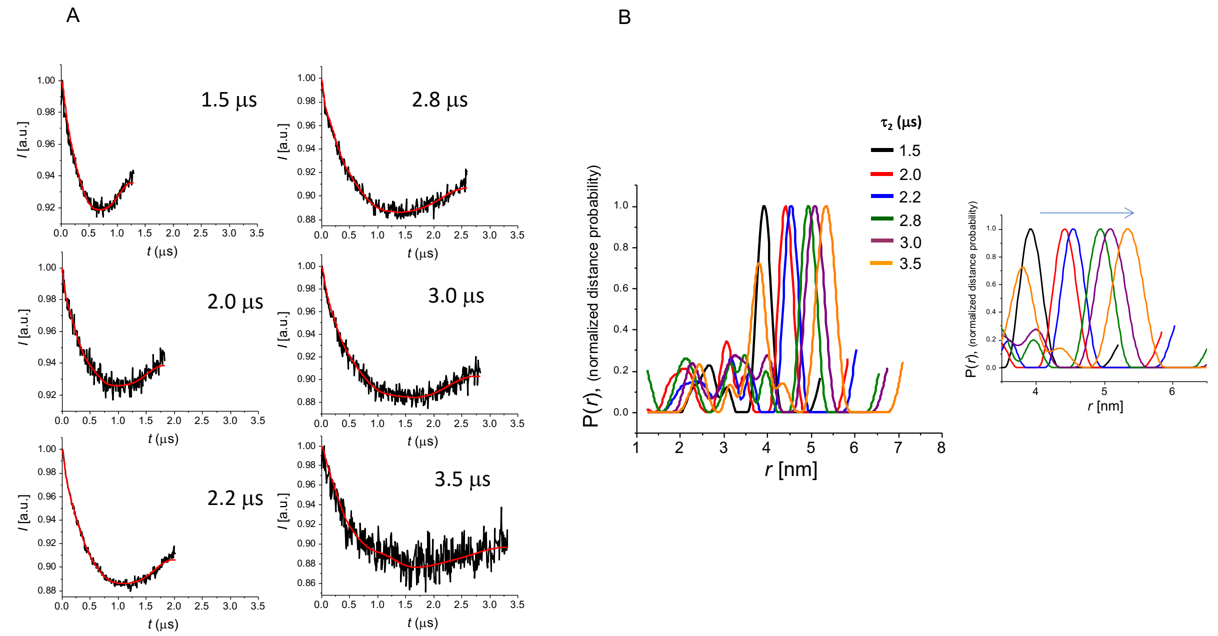

Figure S8. Dipolar evolution signals recorded with different delays τ_2 and corresponding distance distributions. FhaC$^{195R1+503R1}$ in *E. coli* lipids liposomes was used in this experiment. (A) The dipolar evolution signals were measured at increasing dipolar evolution times *t*. (B) The longest distance measured shifts to longer values for longer dipolar evolution times *t* since long, but not short distances are sensitive to the value used in PELDOR experiments. The lipid environment decreases the dipolar evolution time that can be applied, which results in an apparent shift to smaller distance distribution values. The right panel is a zoom on the 4-6 nm region.

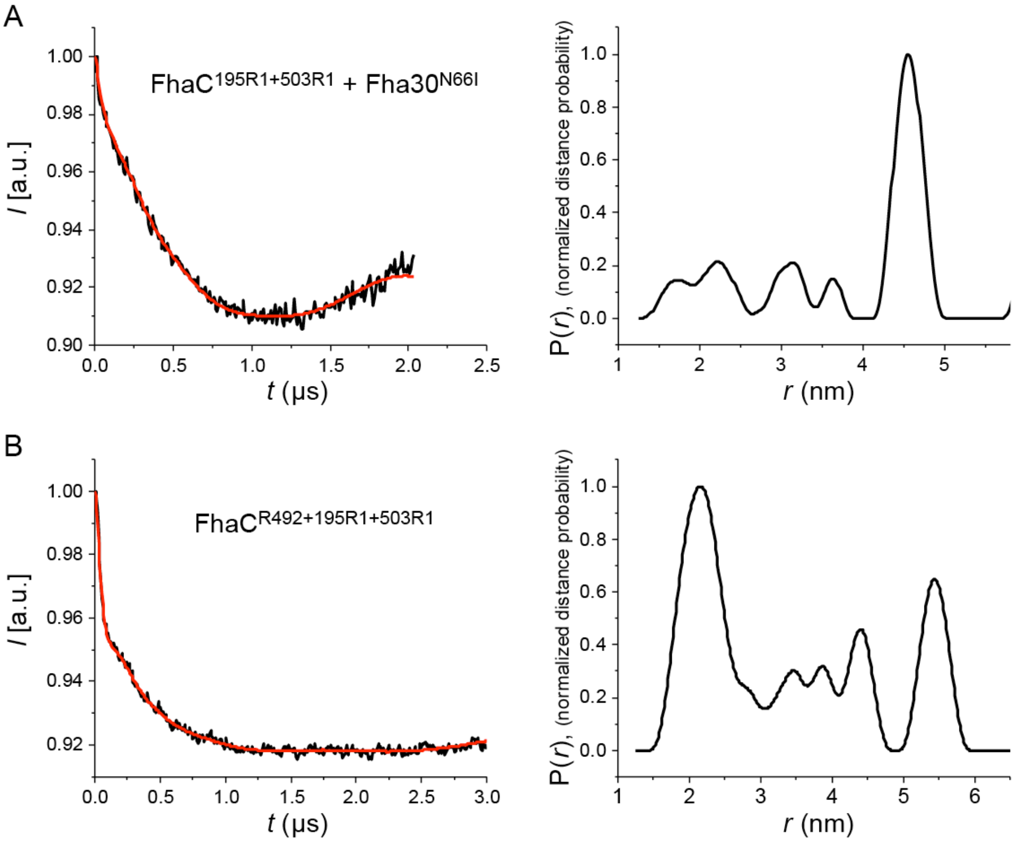

Figure S9. PELDOR analyses of FhaC with substrate and of the FhaC^{R492} mutant. (A). Dipolar evolution function (left) and distance distributions (right) for FhaC$^{195R1+503R1}$ incubated with its substrate, Fha30^{N66I}, in proteoliposomes prepared with *E. coli* polar lipids. (B). Dipolar evolution function (left) and distance distributions (right) for FhaC$^{R492+195R1+503R1}$ in *E. coli* polar lipids proteoliposomes.

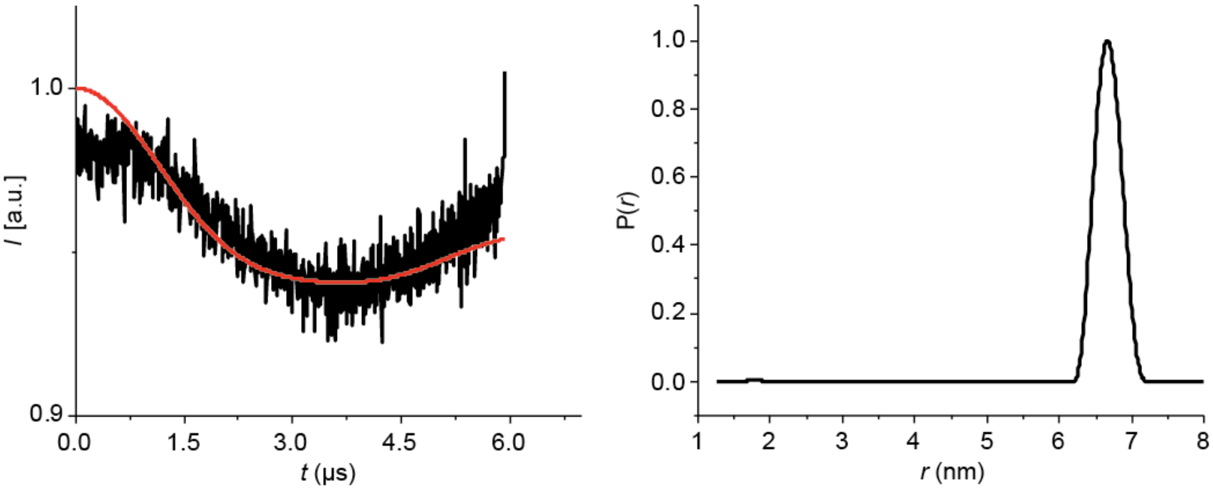

Figure S10. PELDOR analysis of FhaC in nanodiscs. Dipolar evolution function (*left*) and Tikhonov regularization (*right*) of the PELDOR EPR signal of FhaC$^{195R1+503R1}$ in nanodiscs made with the MSP1D1 scaffold protein. Note that similar results were obtained with larger nanodiscs prepared with the MSP1E3D1 protein (not shown).

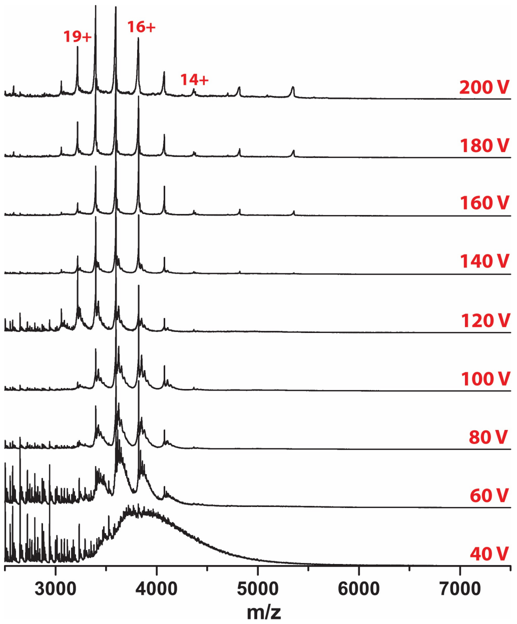

Figure S11. Native MS analysis of FhaC in bOG micelles. The spectra were obtained at increasing collisional energy.

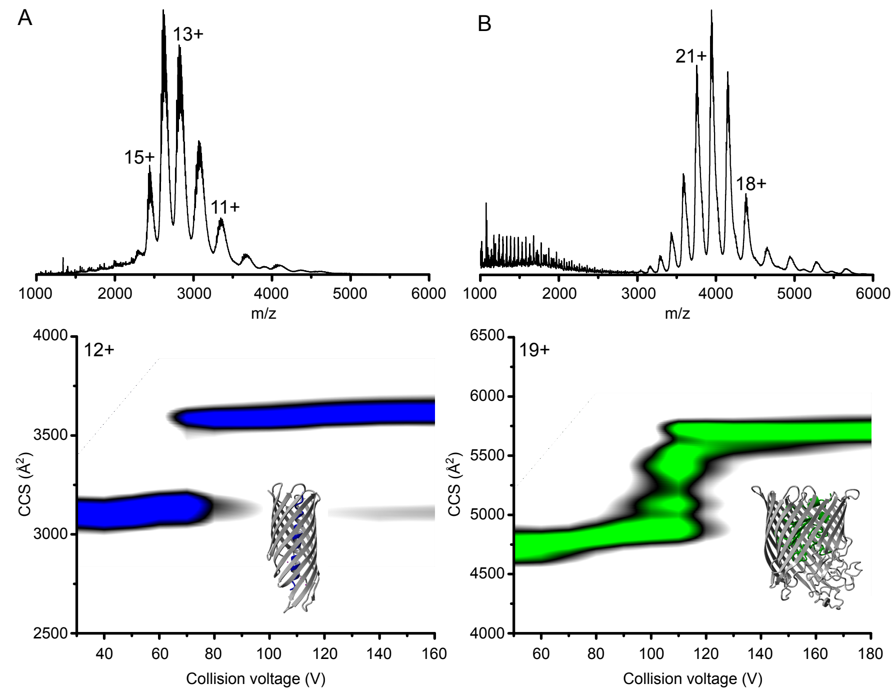

Figure S12. Mass spectra and CIU plots of control OMPs. (A) SphB1-αβ is a truncated autotransporter (AT) containing only the β barrel with the preceding helical linker inserted in the barrel pore. (B) The TonB-dependent transporter BfrG is composed of a β barrel with a soluble N-terminal plug domain inserted in the barrel. The structural models shown are those of related transporters (PDB 1UYN (10) and 3QLB (11), respectively), as the structures of SphB1-αβ and BfrG are not available. The mass spectra of the two OMPs released from their bOG micelles are shown at the top, and the CIU plots are below. Both show a single CIU transition, which suggests that the β barrels remain intact, while the soluble domains are ejected and unfold.

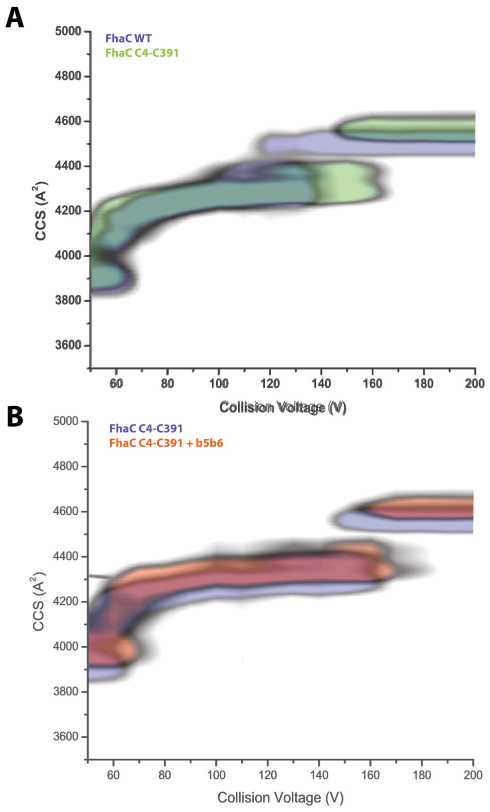

Figure S13. CIU plots of FhaC$^{C4+C391}$. (A) Comparison of the CIU plots of wt FhaC (blue) and the FhaC$^{C4+C391}$ variant (green). (B) Overlay of the CIU plots of unbound FhaC$^{C4+C391}$ (blue) and FhaC$^{C4+C391}$ with the b5-b6 peptide bound (red). As for wt FhaC, binding of the peptide to FhaC$^{C4+C391}$ increased CCS values at both low and high CE, suggesting that it induces enlargement of the β barrel, even with H1 inside.

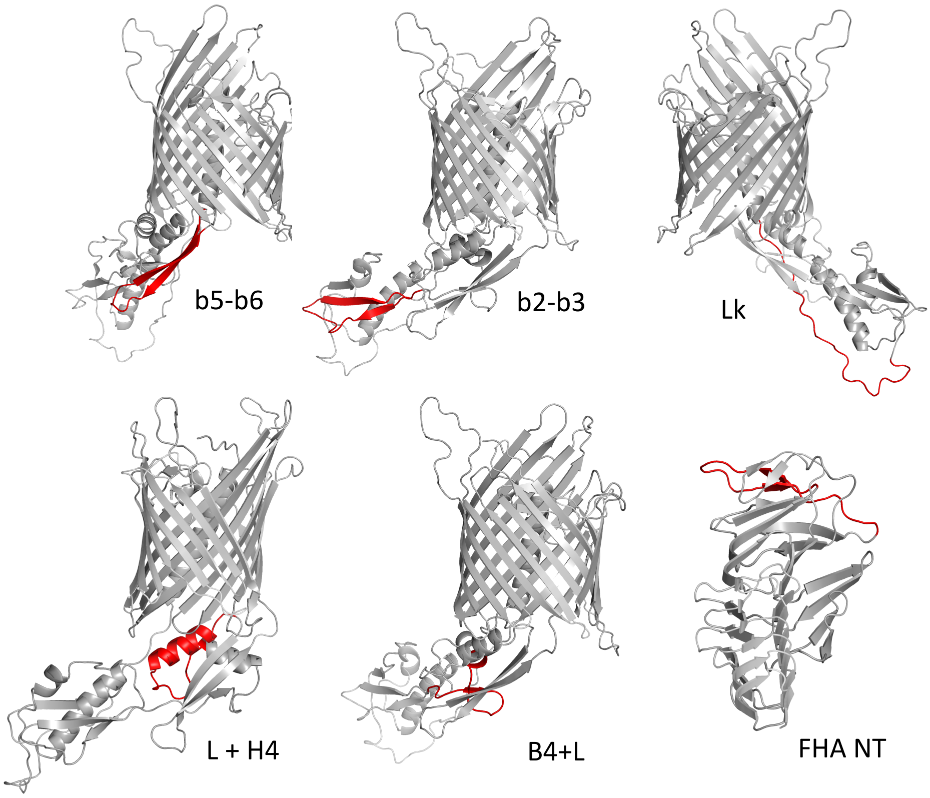

Figure S14. Synthetic peptides used in this study. The first 5 peptides are shown in red on the structural model of FhaC, and the last one on the structural model of the N-terminal portion of FhaB (PDB 1RWR). The b5-b6 peptide (GKTGNITIVPADEYGYSYLDLQLQR) corresponds to the last two β strands of the POTRA2 domain that form an amphipathic β hairpin immediately preceding B1, the first strand of the β barrel. The b2-b3 peptide (SIVTFVPPGVVDGVLKLKVEWGR) encompasses the last two β strands of the POTRA1 domain. It thus corresponds to the same part of the POTRA1 domain as b5-b6 in the POTRA2 domain, and similar to b6, the b3 edge strand of the POTRA1 domain is amphipathic and only slightly shorter than the former. The Lk peptide (RPPVELNPQSEAAAPARKPDATSGH) corresponds to the linker between the H1 helix and the POTRA1 domain. The L+H4 peptide (AMPGWQDKVLNVFDIDQAIYNINNG) encompasses the loop (extended) region that precedes the H4 α helix and the H4 helix of the POTRA2 domain. The B4+L peptide (RIKGWLIDGKPLEGTRDR) corresponds to the β strand b4 of the POTRA2 domain followed by a loop region. Finally, the FHA-NT peptide (QTQVLQGGNKVPVVNIADPNS) corresponds to the N-terminal β strands b2 and b3 of FhaB, preceded and followed by loop regions. All β strands in the TPS domain are very short, and the peptide corresponding to the N-terminal strands β2 and β3 of FhaB was chosen because it forms a hairpin.

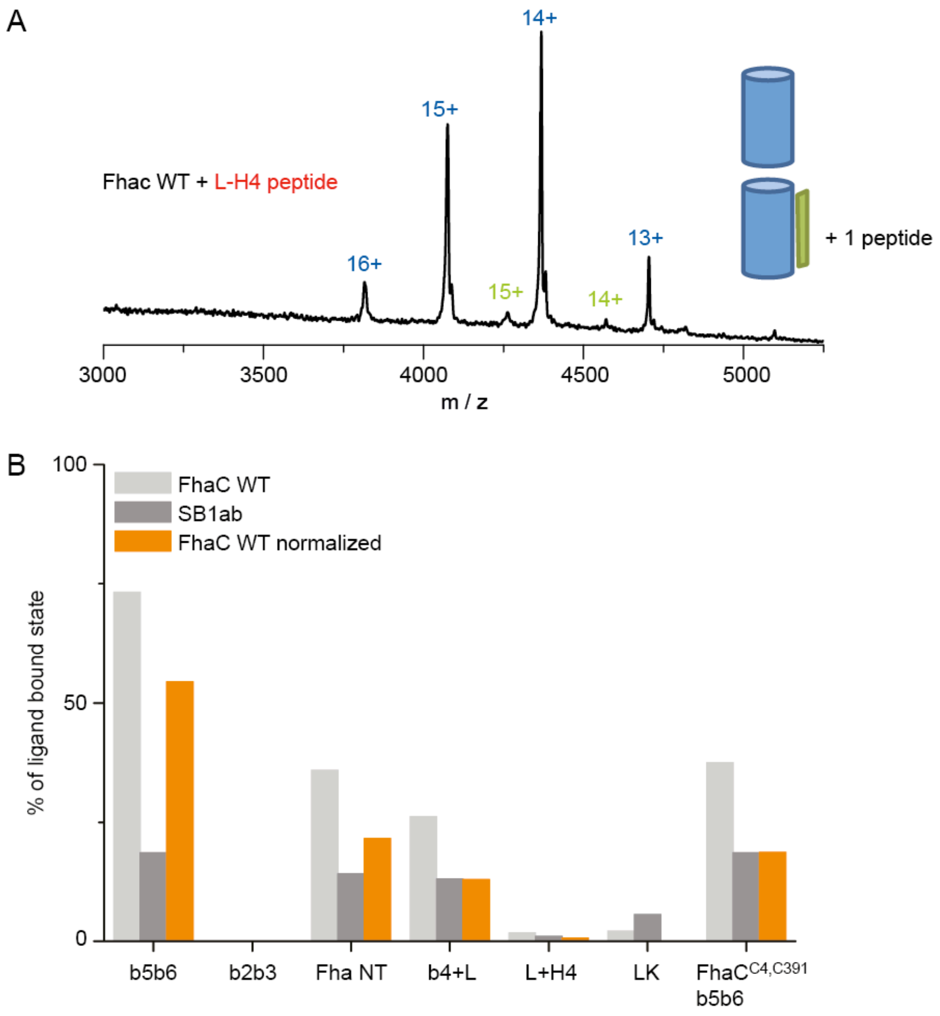

Figure S15. Binding of synthetic peptides to FhaC. (A) Under similar conditions as in Fig. 6A (collisional energy of 150 V), only minimal binding was detected in the mass spectrum of FhaC incubated with the L+H4 peptide. (B) Quantification of the binding of synthetic peptides to FhaC (light grey) and to the control β-barrel protein SphB1-αβ (SB1ab, dark grey; used to correct for non-specific binding). Orange bars show normalized values.

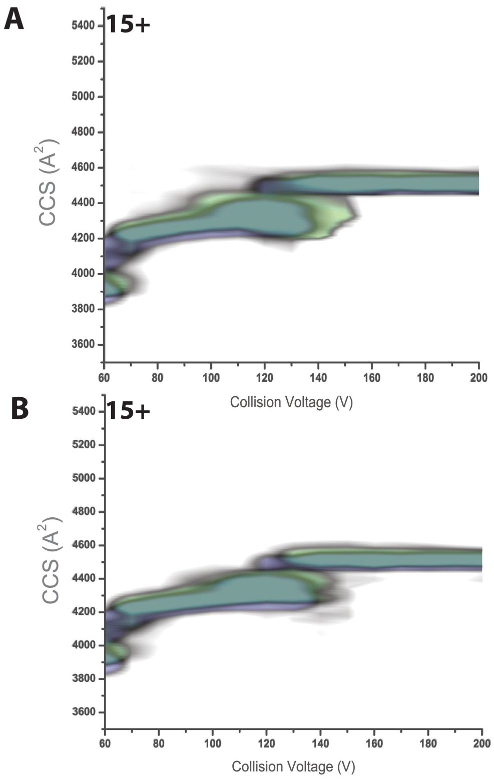

Figure S16. CIU plots of FhaC incubated with synthetic peptides. (A) FhaC without (blue) or with (green) the Fha-NT peptide. (B) FhaC without (blue) or with (green) the B4+L peptide. In both cases the presence of the peptide caused an increased CCS at low collision voltage, but not at elevated collisional activation.

CCS (Å^2)	unbound	+1 peptide	+2 peptides
FhaC WT	3863/4502	-	-
+b5b6	-	3955/4556	4026/4595
+b4L	-	3954/4514	-
+Fha NT	-	3954/4515	-
FhaC C4-C391	3917/4555	-	-
+b5b6	-	3990/4607	ND/4646

Table S17. CCS of FhaC with various peptides determined at low and high CE (listed before and after the slash). The measured values indicate that only b5-b6 enlarges FhaC in both conditions.

References

1. Bennett AE, Griffin RG, Ok JH, Vega S. Chemical shift correlation spectroscopy in rotating solids: Radio frequency-driven dipolar recoupling and longitudinal exchange. J Chem Phys. 1992;96:8624-27.
2. Lewandowski JR, Sass HJ, Grzesiek S, Blackledge M, Emsley L. Site-specific measurement of slow motions in proteins. J Am Chem Soc. 2011;133(42):16762-5.
3. Ma P, Haller JD, Zajakala J, Macek P, Sivertsen AC, Willbold D, et al. Probing transient conformational states of proteins by solid-state R(1rho) relaxation-dispersion NMR spectroscopy. Angew Chem Int Ed Engl. 2014;53(17):4312-7.
4. Hagelueken G, Abdullin D, Schiemann O. mtsslSuite: Probing Biomolecular Conformation by Spin-Labeling Studies. Methods Enzymol. 2015;563:595-622.
5. Hagelueken G, Ward R, Naismith JH, Schiemann O. MtsslWizard: In Silico Spin-Labeling and Generation of Distance Distributions in PyMOL. Appl Magn Reson. 2012;42(3):377-91.
6. Battiste JL, Wagner G. Utilization of site-directed spin labeling and high-resolution heteronuclear nuclear magnetic resonance for global fold determination of large proteins with limited nuclear overhauser effect data. Biochemistry. 2000;39(18):5355-65.
7. Nadaud PS, Helmus JJ, Hofer N, Jaroniec CP. Long-range structural restraints in spin-labeled proteins probed by solid-state nuclear magnetic resonance spectroscopy. J Am Chem Soc. 2007;129(24):7502-3.
8. Lapinaite A, Simon B, Skjaerven L, Rakwalska-Bange M, Gabel F, Carlomagno T. The structure of the box C/D enzyme reveals regulation of RNA methylation. Nature. 2013;502(7472):519-23.
9. Jeschke G. MMM: Integrative ensemble modeling and ensemble analysis. Protein Sci. 2021;30(1):125-35.
10. Oomen CJ, van Ulsen P, van Gelder P, Feijen M, Tommassen J, Gros P. Structure of the translocator domain of a bacterial autotransporter. EMBO J. 2004;23(6):1257-66.
11. Brillet K, Reimmann C, Mislin GL, Noel S, Rognan D, Schalk IJ, et al. Pyochelin enantiomers and their outer-membrane siderophore transporters in fluorescent pseudomonads: structural bases for unique enantiospecific recognition. J Am Chem Soc. 2011;133(41):16503-9.
